# Dynamics of Pathomorphological and Pathophysiological Alterations in Rainbow Trout (*Oncorhynchus mykiss*) During Acute *Aeromonas salmonicida* Infection

**DOI:** 10.3390/biology14101330

**Published:** 2025-09-26

**Authors:** Dmitry Nikiforov-Nikishin, Nikita Kochetkov, Kirill Gavrilin, Viktoria Gaffarova, Kirill Medvedev, Svetlana Smorodinskaya, Anastasia Klimuk, Yuri Kuchikhin, Ivan Svinarev, Natalya Gladysh, Anna Kudryavtseva, Egor Shitikov, Alexei Nikiforov-Nikishin

**Affiliations:** 1 Faculty of Biotechnology and Fisheries, Moscow State University of Technologies and Management (FCU), 73, Zemlyanoy Val Str., 109004 Moscow, Russia; niknikdl@rambler.ru (D.N.-N.); gaff-vik@mail.ru (V.G.); kler.smo@gmail.com (S.S.); klimukanastasia27@gmail.com (A.K.); kuchikhin@gmail.com (Y.K.); 9150699@mail.ru (A.N.-N.); 2 National Fund for Environmental Protection and Development of the Far North and Equivalent Territories “Yakutia”, 18 Oktyabrskaya Street, 677027 Yakutsk, Russia; k.gavrilin@yandex.ru; 3 Division of Biomedicine and Genomics, Lopukhin Federal Research and Clinical Center of Physical-Chemical Medicine of Federal Medical Biological Agency, 119435 Moscow, Russia; mke2341@gmail.com (K.M.); eshitikov@mail.ru (E.S.); 4 LLC “Inarctica NW”, 7 Kominterna Street, 183038 Murmansk, Russia; svinarev@inarctica.com; 5 Engelhardt Institute of Molecular Biology, Russian Academy of Sciences, Vavilov Str., 32, 119991 Moscow, Russia; natalyagladish@gmail.com (N.G.); rhizamoeba@mail.ru (A.K.)

**Keywords:** aquaculture, septicemia, bacteremia, histopathology, hematology, blood biochemistry, pathomorphology, immune response, neutrophilia, virulence

## Abstract

Furunculosis caused by *Aeromonas salmonicida* is one of the most common diseases in aquaculture, leading to significant economic losses. This study comprehensively investigated the dynamics of pathophysiological and histopathological disorders in rainbow trout (*Oncorhynchus mykiss*) infected with the moderately virulent strain *A. salmonicida* SL0n. Whole-genome analysis showed that strain SL0n belongs to the *A. salmonicida* species complex, possessing a single circular chromosome. The genome encodes a wide range of virulence factors, including adhesion systems (type IV pili, fimbriae), toxins (aerolysin, hemolysins), and a type II secretion system (T2SS), but notably lacks plasmids and a type III secretion system (T3SS). This genomic profile likely dictates a pathogenic mechanism reliant on secreted exotoxins (via T2SS), explaining the observed systemic cytotoxic damage. In an acute experiment, the 4-day LD_50_ was determined to be 1.63 × 10^6^ CFU/fish. In a prolonged experiment, fish were injected with a sublethal dose (1.22 × 10^6^ CFU/fish—75% of LD50). The disease progressed through three consecutive stages. The early stage (1–2 DPI) was characterized by maximal bacterial load and activation of nonspecific immunity. The acute stage (4 DPI) manifested as severe septicemia and anemia, associated with systemic organ damage, which correlated with peak AST and ALT enzyme activity. The recovery stage (6 DPI) was marked by partial regression of inflammation, key biochemical and histological parameters indicated persistent liver and kidney dysfunction, signifying an incomplete recovery. These results demonstrate the pathogenesis of acute furunculosis and reveal that the genomic profile of the SL0n strain causes a sequential, systemic infection characterized by severe organ dysfunction.

## Simple Summary

Bacterial diseases in fish are a primary cause of mortality in global aquaculture. This study presents experimental data on the pathogenesis in rainbow trout infected with a moderately pathogenic strain of *Aeromonas salmonicida*. The study determined the dose causing 50% mortality (LD50) and documented associated clinical and pathological abnormalities. As part of a prolonged experiment, the disease course was examined following infection with a sublethal dose of the pathogen, and physiological and histological abnormalities were compared on days 1–6 post-infection. The findings revealed a relationship among the microbiological load, clinical signs, physiological indicators, and histopathological changes during the acute form of the disease. This combination of factors resulted in significant organ dysfunction and an incomplete recovery, with persistent signs of pathology.

## 1. Introduction

Furunculosis is one of the most common infectious diseases of fish in aquaculture, owing to its high prevalence and ability to infect a wide range of hosts [1,2,3]. Traditionally, the non-motile aeromonad *Aeromonas salmonicida* is considered the sole etiological agent of furunculosis, often without specifying a particular subspecies. The subspecies *A. salmonicida* ssp. *salmonicida* is recognized as the typical pathogen causing high mortality, mainly in salmonid species [2]. However, other so-called “atypical” subspecies of *A. salmonicida*, such as *achromogenes*, *masoucida*, *smithia*, and *pectinolytica* [4], have also been isolated from various infected fish species exhibiting diverse clinical symptoms [5,6,7,8].

The virulence of *A. salmonicida* isolates varies significantly, depending on geographical origin, host-species specificity, and the presence of virulence factors [9,10,11]. In particular, *A. salmonicida* ssp. *salmonicida* has been repeatedly isolated from clinically healthy fish [12,13]. The diversity of clinical manifestations of furunculosis is thought to depend on several key factors: strain virulence, culture conditions, the physiological state of the fish, and individual genetic resistance [10,14,15,16]. Fish that survive a bacterial infection develop long-term immunity. However, the significant diversity of strains and serotypes has hindered the development of a universal vaccine providing cross-protective immunity [17,18,19]. In addition, *Aeromonas spp*. often develop antimicrobial resistance, largely due to the intensive use of antibiotics [20]. The effectiveness of furunculosis therapy depends not only on the efficacy of the antibiotic used, but also on the timeliness of treatment initiation. In the ulcerative course of the disease, even successful therapy leads to a significant decrease in the growth rate of surviving individuals and a decline in their commercial quality [21]. Developing more effective therapeutic strategies requires considering not only the virulence of a particular strain, but also its prevalence in the aquatic environment and host organism, as well as its transmission routes. Furthermore, selective breeding has been successfully used to obtain lines of various fish species resistant to furunculosis [22,23].

According to current understanding, the virulence of *A. salmonicida* is determined by a complex set of factors: adhesion factors, extracellular hydrolytic enzymes, exotoxins, secretory systems, and lipopolysaccharides [10,24,25]. These factors allow the pathogen to attach, colonize, invade, multiply, and damage host cells, leading to the development of the disease [26]. The activity of these factors can vary both with environmental conditions (e.g., host species, microbial community composition, water quality) [25,27] and during isolation and cultivation on nutrient media [28,29].

Furunculosis is characterized by a variety of clinical and pathomorphological manifestations [10,30,31,32]. Furunculosis presents in three forms: subacute, acute, and chronic. The subacute form is associated with a relatively favorable outcome. The acute form manifests as severe septicemia and ulceration, leading to mass fish mortality. Finally, the chronic form is characterized by less pronounced clinical signs and a persistent infection. Typical clinical signs include anorexia, swimming disorders, hemorrhages on the skin and internal organs, exophthalmos, and ascites [5,33,34]. In natural outbreaks on fish farms, mass mortality can be observed within 15–30 days in the chronic form and within 2–5 days in the acute form, but the rate of disease development is influenced by water quality, age, and the innate resistance of the fish [34,35,36]. In experimental infection, pathological changes develop more rapidly, but their rate depends on the dose and route of inoculation/infection (oral, intramuscular, intraperitoneal, immersion) [37,38]. Of particular interest are studies that describe the pathogenesis caused by various strains of non-motile aeromonads, along with their biochemical and genetic characteristics. The study of moderately virulent strains may overcome the limitations of research using highly lethal isolates, which often result in rapid mortality that precludes a detailed temporal analysis of the host–pathogen interaction.

Despite a significant number of studies on the experimental infection of fish with *A. salmonicida*, data on strain virulence, typically expressed as the median lethal dose (LD50), remain inconsistent [39,40,41]. In this regard, the study of the pathogenicity of individual *A. salmonicida* strains is particularly relevant, as this will help build a unified understanding of the disease’s pathogenesis and provide a basis for identifying clinical markers to develop effective therapeutic and preventive strategies.

A detailed study of the pathogenesis of bacterial diseases requires an assessment of the dynamics of the pathological process based on a set of physiological and morphological indicators. Despite the active development and implementation of molecular biological technologies in aquaculture, the study of disease pathogenesis based on classical clinical and morphological approaches remains of fundamental importance. A comprehensive study of the clinical picture, hematological changes, biochemical markers, and histological indicators provides a holistic view of the pathogenesis at the whole-organism level. Histopathological studies are an important tool that provides valuable information about changes at the tissue and cellular levels in different organs affected by pathogen invasions [42,43]. While the pathogenesis of septicemia caused by motile aeromonads has been described in detail for both salmonids [44] and other fish species [33,45], a comprehensive approach for identifying the relationship between the clinical picture and the degree of morphophysiological disorders in organs and tissues has not yet received sufficient attention.

The aim of this study was to characterize, under experimental conditions, the relationship between clinical signs, biochemical and morphological blood parameters, and pathomorphological changes in tissues in rainbow trout (*Oncorhynchus mykiss*) infected with sublethal doses of *Aeromonas salmonicida* strains with established virulence factors.

## 2. Materials and Methods

### 2.1. Fish Housing Conditions

One thousand rainbow trout (*Oncorhynchus mykiss*, 8 months old, 18.5 ± 0.5 cm and 85.3 ± 8.0 g) were obtained from the fish farm LLC Rusregionbiznes (Kondrovo, Russia). The fish were then acclimatized for 28 days in a recirculating aquaculture system (RAS). The RAS included a 4000 L tank equipped with biological (1000 L biofilters) and mechanical filtration systems (Eiskpolimer 402M, Eiskpolimer, Eisk, Russia), as well as ultraviolet installations (UOV-PV-5 in ECO-1A105H40US version, Alexandra-Plus LLC, Vologda, Russia). The system’s water exchange rate was 70 L/h, with 8% fresh water added daily. The water temperature in the fish tanks during cultivation was 16.05 ± 0.2 °C, the pH was 7.46 ± 0.05, and the oxygen content was 9.1 ± 0.2 mg/L. The content of nitrogenous metabolites and phosphates was within the normal range for this species. Fish were fed twice daily (at 11:00 and 17:00) with commercial granular feed (Trout Growth 44/23 A75, GK MELKOM, Tver, Russia), according to the feeding tables provided by the manufacturer.

### 2.2. Ethics Statement

This study was conducted in accordance with the guidelines of the Local Ethics Commission of the Scientific and Technical Council of the Moscow State University of Technology and Management (approval number 1, 16 January 2025) and in accordance with the Guidelines for the Care and Use of Laboratory Animals of the National Institutes of Health.

### 2.3. Bacterial Isolation, Physiological and Biochemical Tests

The isolate, identified as *Aeromonas salmonicida*, was collected from the water of the RAS (Moscow, Russia) in April 2025. The isolate was incubated on nutrient agar (NA) (State Research Center of Applied Microbiology and Biotechnology (SRCAMB), Obolensk, Moscow Region, Russia) for 24 h at 25 °C, followed by serial dilutions to obtain a pure culture. The pure bacterial culture was stored in ultralow temperature freezer at −80 °C (DW-HL340, Meling, Hefei, China) in tryptic soy broth (TSB) with 20% (*v*/*v*) glycerol added.

Gram staining and determination of biochemical characteristics (oxidase, Voges-Proskauer test, OF test, H_2_S formation, indole formation, catalase, lactose fermentation, glucose utilization) were performed in accordance with standard microbiological procedures described in Bergey’s Manual [46]. The effect of NaCl concentration on the growth of the isolate was assessed by transferring the culture to fresh nutrient medium with the addition of NaCl in various concentrations (from 1% to 2%). The effect of temperature on culture growth was evaluated at 13, 17, 27, and 37 °C using a thermostat (Binder 115, Binder GmbH, Tuttlingen, Baden-Württemberg, Germany). All experiments were performed in three independent replicates (*n* = 3).

Antibiotic sensitivity was analyzed using the disk diffusion method developed by Bauer et al. [47] with indicator disks (SRCAMB, Moscow Region, Russia): gentamicin (10 µg); bacitracin (0.04 units); ciprofloxacin (5 µg); ceftriaxone (30 µg); chloramphenicol (15 µg); azithromycin (15 µg); and cefixime (5 µg). The sensitivity of the strain was determined by the size of the growth inhibition zone in accordance with the recommendations of Whitman [48].

Fish blood agar (FBA) was prepared using blood collected from healthy rainbow trout. Blood collection was performed under aseptic conditions using sterile syringes (2 mL, 24G needle) pretreated with sodium heparin solution (5000 IU/mL) to prevent coagulation. The blood was added to sterile GRM-1 Agar (composition (g/L): Pancreatic hydrolysate of fish meal, 12.0; Enzymatic peptone, 12.0; Sodium chloride, 6.0; Microbiological agar, 10.0 ± 2.0) (SRCAMB, Obolensk, Russia) to a final concentration of 5% (*v*/*v*). The prepared medium was poured into Petri dishes and stored at 4 °C until use. The sterility of the medium was checked by incubating several dishes at 25 °C for 24 h; no microbial growth was observed. To prepare the inoculum, the *A. salmonicida* strain was cultured on GRM-1 agar (without blood) at 25 °C for 24 h. The resulting biomass was suspended in 10 mL of sterile saline solution. The suspension was standardized by optical density to match the McFarland 1.0 turbidity standard, and then it was serially diluted to 1:100,000. FBA plates were inoculated with the resulting inoculum and incubated at 25 °C for 24 h, after which the presence of hemolysis zones was assessed.

### 2.4. Genome Sequencing

Genomic DNA was extracted from an overnight culture of *A. salmonicida* SL0n (OD_620_ = 0.6) grown in Luria–Bertani broth using the Wizard Genomic DNA Purification Kit (Promega, Madison, WI, USA) according to the manufacturer’s instructions. DNA concentration and purity were measured with a Qubit 4 Fluorometer and a Nanodrop ND-1000 spectrophotometer (Thermo Fisher Scientific, Waltham, MA, USA). For library construction, 100 ng of purified genomic DNA was processed with the KAPA HyperPlus Kit (Roche, Basel, Switzerland) following the manufacturer’s protocol. The library was purified using KAPA HyperPure Beads (Roche, Basel, Switzerland). Library size distribution and integrity were assessed with a High Sensitivity DNA chip (Agilent Technologies, Santa Clara, CA, USA), and quantification was performed using the Quant-iT DNA Assay Kit, High Sensitivity (Thermo Fisher Scientific).

DNA libraries were sequenced on the NextSeq 1000 platform (Illumina Inc., San Diego, CA, USA) following the manufacturer’s specifications. The NextSeq 1000/2000 P2 Reagents kit (200 Cycles) v3 was employed in conjunction with a 2% PhiX spike-in control.

### 2.5. Genome Assembly and Bioinformatics Analysis

Quality assessment of paired-end reads was conducted using FastQC v0.12.1 [49]. Adapter trimming and read filtering were performed with fastp v0.23.4 [50]. Taxonomic verification and contamination screening used BLAST v2.17.0 [51] analysis on 100 randomly selected reads. Genome assembly was accomplished using Unicycler v0.5.1 [52]. The draft genome assembly was annotated using the NCBI Prokaryotic Genome Annotation Pipeline (PGAP) [53]. This Whole Genome Shotgun project has been deposited at DDBJ/ENA/GenBank under the accession JBQCCD000000000. The version described in this paper is version JBQCCD010000000.

Genome-based taxonomic classification was performed using the Type Strain Genome Server (TYGS) on the DSMZ platform (http://ggdc.dsmz.de/, accessed 12 August 2025) [54]. Additionally, average nucleotide identity (ANI) values were calculated employing BLASTN-based (ANIb) and MUMMER-based (ANIm) algorithms via the JSpeciesWS tool (https://jspecies.ribohost.com/jspeciesws/, accessed 12 August 2025) [55].

Plasmid detection was undertaken using PlasmidFinder v2.1 [56] with the threshold established at 80%. Prophage sequences were predicted employing the PHASTER web server [57]. Transposon and insertion sequence identification was conducted using TnCentral BLAST [58]. Virulence-associated genes were detected utilizing VF Analyzer based on the Virulence Factor Database (VFDB) [59], with the detection threshold set at 90%. Antimicrobial resistance genes were identified using the Resistance Gene Identifier (RGI v6.0.5) from the Comprehensive Antibiotic Resistance Database (CARD v4.0.1) [60], applying default parameters. All aforementioned tools were accessed on 13 August 2025.

### 2.6. Experimental Infection

**Figure 1:**
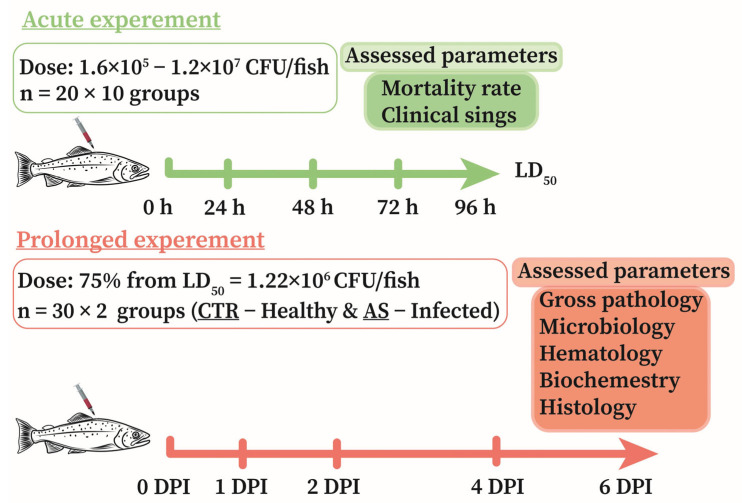
Scheme of experiments to determine the dynamics of pathological disorders in rainbow trout infected with *A. salmonicida* SL0n.

To study the dynamics of pathological changes induced by the isolated strain *A. salmonicida* SL0n, two series of experiments were conducted (Figure 1): (I) an acute experiment to determine the LD50 and comprehensively assess virulence; and (II) a prolonged experiment to identify the stages of disease development (pathogenesis) under the influence of a sublethal dose (75% of LD50). For these studies, rainbow trout without visible signs of disease or injury, measuring 21.1 ± 1.1 cm and weighing 141.2 ± 10.5 g, were selected.

Acute and prolonged experiments were conducted in recirculating aquaculture systems (RAS) comprising 700 L tanks, a mechanical filter (GREEN RESET 25–40, Sicce S.r.l., Pozzolone, Italy), a 500 L biofilter, and UV units (Jebao STU-40w, Zhongshan, China). Water replacement in the system was 6% per day. The temperature, oxygen content, and other hydrochemical parameters within the systems corresponded to those in the holding tanks. Control and experimental fish were kept in different tanks.

To obtain bacterial biomass, *A. salmonicida* SL0n culture was grown on GRM-1 Agar solid medium for 24 h at 25 °C under aerobic conditions. The grown biomass was washed off the agar surface with sterile phosphate-buffered saline (PBS, pH 7.2–7.4). To remove residual nutrient medium and metabolites, the resulting suspension was washed three times. Each washing cycle included centrifugation at 4000× *g* for 10 min at 4 °C (M1324R, RWD Life Science, Shenzhen, China), followed by supernatant removal and resuspension of the bacterial pellet in sterile PBS. Cell concentration in the suspension was assessed spectrophotometrically by measuring the optical density (OD) at a wavelength of 600 nm (OD_600_). The initial suspension was diluted with sterile PBS to achieve working concentrations. The number of viable cells in the working suspension was confirmed by plate counting on GRM-1 Agar. Only freshly prepared suspensions with confirmed bacterial concentrations were used for the infection experiment.

To determine the LD50 (acute experiment), a range of doses per individual was selected, considering the significant variability in virulence among different isolates of *A. salmonicida*: 1.6 × 10^5^, 3.2 × 10^5^, 6.5 × 10^5^, 1.5 × 10^6^, 3.0 × 10^6^, 6.0 × 10^6^, 8.0 × 10^6^, 1.0 × 10^7^, and 1.2 × 10^7^ CFU/fish. This dose range was established based on results from preliminary experiments and an analysis of literature data [37,61,62]. Ten experimental groups, each comprising 20 rainbow trout (*n* = 20), were formed: one control group, injected intramuscularly (IM) with 0.2 mL of PBS per individual, and nine experimental groups, IM injected with 0.2 mL of *A. salmonicida* SL0n bacterial suspension containing the corresponding amount of bacteria. During the 4-day acute experiment, fish survival, behavioral changes, and the development of clinical signs were assessed daily.

The sublethal dose of *A. salmonicida* determined in the acute experiment was used in the prolonged study. Two groups, each comprising 30 rainbow trout (*n* = 30), were formed for the prolonged experiment: (I) Control group (CTR): similar to the control group in the acute experiment (PBS injection); (II) Experimental group (AS): injected with *A. salmonicida* SL0n at a dose of 1.22 × 10^6^ CFU/fish (75% of LD50). The prolonged experiment lasted 6 days. On days 0, 1, 2, 4, and 6 post-injection (DPI), a comprehensive assessment of fish condition was performed for both groups (*n* = 6), including: clinical signs of disease; microbiological load; hematological and biochemical blood analyses; and histological examination of the muscles, intestines, liver, kidneys, and spleen.

On the first day of the prolonged experiment, no significant differences in the severity of clinical signs were observed between the control and experimental groups. Therefore, individuals for biomaterial collection were selected randomly at this time point. At subsequent time points (2, 4, and 6 DPI), individuals with the most pronounced clinical picture were selected to assess the dynamics of pathological changes. We acknowledge that this non-random sampling introduces a selection bias and may overestimate the severity of the disease. However, this approach to individual selection was driven by the need to study the dynamics of pathological changes at different stages of the disease, as, according to the literature, the manifestation of obvious clinical signs in fish usually precedes death [34]. Given the individual sensitivity of fish to the disease, random individual selection would lead to high sample heterogeneity in the measured parameters.

### 2.7. Blood Sampling and Hematological Study

Fish were anesthetized by immersion in MS-222 solution at a concentration of 20 mg/L until loss of equilibrium was observed. Blood samples (1.5 mL) were collected from the caudal vein using sterile 2 mL syringes with 24 G needles. Blood was collected from six fish (*n* = 6) from each experimental group at five time points: 0, 1, 2, 4, and 6 DPI. The total number of erythrocytes and leukocytes was determined immediately after sampling using a hemocytometer according to the method described by Blaxhall and Daisley [63]. Peripheral blood smears were prepared using a standard method [64]. The smears were stained with Romanovsky-Giemsa (Ecolab, Moscow, Russia).

Differential blood cell counts were performed on prepared smears, including erythrocytes and their immature forms, lymphocytes, monocytes, neutrophils (segmented and band), basophils, and thrombocytes. For each preparation, at least 50 fields of view were examined, counting a minimum of 7000 erythrocytes and 150 leukocytes. Differential leukocyte counts were expressed as a percentage of the total number of leukocytes. The counting and identification of blood cells were performed according to established morphological criteria [65,66,67].

### 2.8. Bacterial Load Assessment

To determine the bacterial load in fish, aseptic samples of liver and skeletal muscle tissue, each weighing approximately 5 g, were taken. The muscle tissue sample collected from the contralateral side. Each sample was placed in a pre-weighed sterile test tube containing 5 mL of sterile phosphate-buffered saline (PBS, pH 7.2). The test tubes with tissue samples were reweighed on analytical scales to accurately determine the mass of each sample. The tissues were homogenized using a homogenizer (MT-13K-L, MIULAB, Hangzhou, China). For quantitative analysis, a series of tenfold dilutions in PBS were prepared from the homogenate. Plating on Endo Agar (SRCAMB, Obolensk, Russia) was performed using the surface method. Fifty microliters of whole blood, as well as each corresponding dilution of tissue homogenates, were evenly distributed over the surface of the agar using a sterile spatula. The cultures were incubated in a thermostat at 25 °C for 24 h. After incubation, the number of colony-forming units (CFU) on the plates was counted. The bacterial load was expressed in CFU per 1 mL of blood (CFU/mL) or per 1 g of tissue (CFU/g), taking into account the mass of the original sample and the degree of dilution.

To confirm species identification, confirmatory testing was performed on 3–5 colonies from each plate. All tested isolates showed the same phenotype consistent with that of *A. salmonicida* SL0n: oxidase (+), catalase (+), non-motile, and fermented glucose with acid and gas production (O+/F+).

### 2.9. Blood Biochemical Parameters

The collected blood was placed in vacuum tubes (MiniMed, Suponevo, Russia) and incubated at 4 °C for 15–30 min for coagulation. After coagulation, the blood was centrifuged at 5000× *g* rpm for 10 min (DM0506 centrifuge, Dlab Scientific, Beijing, China) to separate the serum. Serum samples were analyzed for substrate content (total protein, albumin, globulin, total bilirubin, direct bilirubin, creatinine, glucose, urea) and enzymes (alkaline phosphatase, alanine aminotransferase, aspartate aminotransferase, and lactate dehydrogenase) using an automatic biochemical analyzer CS-T240 (DIRUI, Changchun, China) with ready-made reagent kits supplied by DIRUI, following the manufacturer’s instructions.

### 2.10. Histological Study

After blood sampling, six fish (*n* = 6) from each experimental group were dissected at five time points to collect tissue from the following organs: muscle at the injection site, liver, spleen, hindgut, and body kidney. Tissue samples weighing 3–7 g were fixed for 24 h in Davidson’s solution, then transferred to 70% ethanol for further processing. The tissue samples were dehydrated through a series of increasing ethanol concentrations (80% for 1 h, 95% for 1 h, and 99% for two 1 h periods) (Citadel 2000, Thermo FS, Waltham, MA, USA). Subsequently, the samples were cleared in xylene for 1.5 h and embedded in paraffin. Three serial microtome sections (Minux S700A, RWD Life Science, Shenzhen, China) with a thickness of 4 μm were prepared from the samples and stained with hematoxylin-eosin (H&E), periodic acid-Schiff (PAS), and Masson’s trichrome (MT) (Biovitrum, St. Petersburg, Russia). The preparation and staining of histological sections were performed according to the generally accepted method [68]. The resulting preparations were viewed under an Olympus BX53 light microscope (Olympus Corp., Tokyo, Japan), equipped with Carl Zeiss ERc 5s eyepiece attachments (Zeiss, Oberkochen, Baden-Württemberg, Germany) and a ToupCam 16.0 MP camera (ToupTek Photonics, Hangzhou, China). Images were captured and processed using ZEN lite (Zeiss) and ToupCam view 16.0 (ToupTek Photonics) software. Adobe Photoshop 2024 (Adobe Systems, San Jose, CA, USA) was used to adjust image parameters such as brightness, contrast, and lighting balance.

### 2.11. Histomorphometric Analysis

Histomorphometric parameters were measured using Fiji ImageJ2 v2.15.0 software [69]. The measured parameters for each tissue are listed in Table S1. The selection and measurement methods for these parameters followed previous studies [70,71] and other published data [72,73].

### 2.12. Statistical Analysis

The comparison data for the analyzed variables are presented as means ± SD. Statistical significance between experimental groups or sampling times was determined using one-way ANOVA with Tukey’s post hoc test or Kruskal–Wallis with Dunn’s post hoc test, depending on the distribution of data and homogeneity of variance (Shapiro–Wilk and Levene tests). A *p*-value < 0.05 was considered statistically significant. The median lethal concentration (LD50) was calculated graphically using probit analysis [74]. The 95% confidence intervals (CI) for the obtained LD50 values were also determined. Statistical data were processed using GraphPad Prism v 9.0 (GraphPad, San Diego, CA, USA) and RStudio v 3.6.0 (Posit, PBC, Boston, MA, USA) [75], which uses the R v 4.4.3 programming language (R Core Team, Vienna, Austria, 2025) [76].

## 3. Results

### 3.1. Characterization of the A. salmonicida Isolate

**Figure 2:**
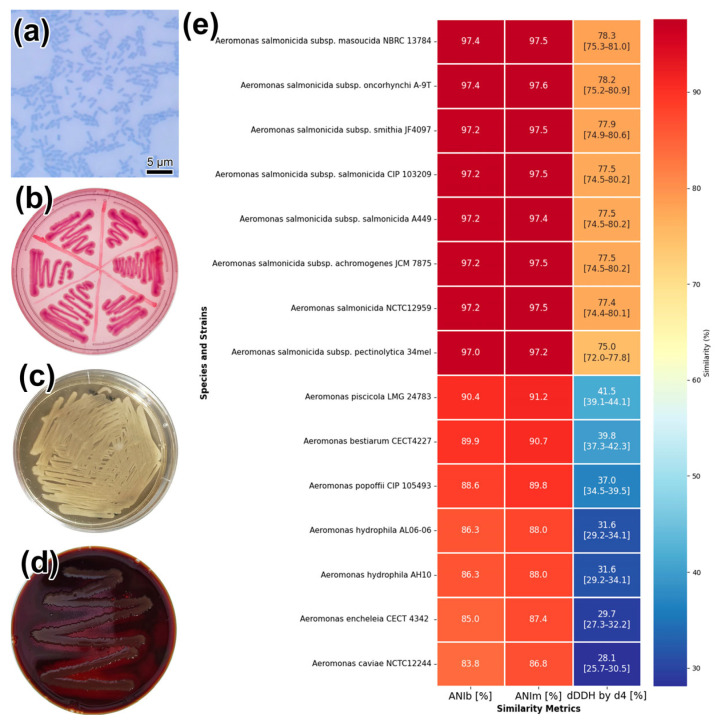
Phenotypic and genotypic characteristics of Aeromonas salmonicida SL0n strain: (**a**) Microscopy of cells after staining with methylene blue (scale bar 5 μm); (**b**) colony morphology on Endo agar, (**c**) GRM1, and (**d**) FBA media; (**e**) Heatmap visualization of genomic similarity between A. salmonicida SL0n and various Aeromonas species. The matrix shows pairwise similarity percentages with a color gradient from dark blue (low similarity) to dark red (high similarity), providing a clear view of genetic relationships. Numeric values indicate the measured similarity percentages. Square brackets within the dDDH by d4 column represent the corresponding confidence intervals for each measurement.

The results of phenotypic and biochemical analysis showed that the isolated strain is a Gram-negative, non-motile, oxidase- and catalase-positive rod (Figure 2a; Table S2). On Endo agar, the strain formed small, pink, round, glossy colonies (Figure 2b); on GRM1 medium, it formed cream-colored, round colonies (Figure 2c). Growth was rapid; in primary and subsequent cultures at 25 °C, visible colonies formed within 24 h. The strain fermented glucose to form acid and gas but did not utilize lactose. The reactions for indole, H_2_S, and the Voges-Proskauer test were negative. The isolate was capable of growing in the temperature range of 13–37 °C and in the presence of 0–2% NaCl. Using the disk diffusion method, the strain showed sensitivity to ciprofloxacin, gentamicin, chloramphenicol, and azithromycin but was resistant to bacitracin, cefixime, and ceftriaxone (Table S2). When cultured on blood agar, the isolate’s colonies produced a characteristic brown, water-soluble pigment and exhibited a pronounced zone of β-hemolysis (observed as a zone of discoloration) (Figure 2d).

### 3.2. Genome Characteristics and Taxonomic Analysis

The draft genome assembly of strain SL0n comprised a 4,707,138 bp circular chromosome with an average GC content of 58.42%. The assembly has an N50 value of 349,787 bp with sequencing coverage of 101-fold. Annotation by the NCBI Prokaryotic Genome Annotation Pipeline (PGAP) analysis identified a total of 4397 genes, including 4289 coding DNA sequences (CDS), of which 4233 encoded putative protein products.

Taxonomic assessment via the Type Strain Genome Server (TYGS) revealed that strain SL0n clustered robustly within the *Aeromonas salmonicida* spp. clade. Genome Blast Distance Phylogeny (GBDP) using formula d4 did not resolve subspecies boundaries, suggesting the possibility of a novel subspecies. Digital DNA–DNA hybridisation (dDDH) yielded highest values with *A. salmonicida* subsp. *masoucida* NBRC 13784T (78.3%) and subsp. *oncorhynchi* A-9T (78.2%) (Figure 2e). The lowest intraspecies dDDH value was observed with *Aeromonas salmonicida* subsp. *pectinolytica* DSM 12,609 (75.2%). As the species threshold for dDDH is 70%, these data support assignment of SL0n to the *A. salmonicida* species complex.

Comparative average nucleotide identity (ANI) analysis corroborated this placement (Table 1). The ANIb values ranged from 96.59% to 97.27%, whilst ANIm values spanned 97.22% to 97.58%. The highest sequence similarities were observed with *A. salmonicida* subsp. *masoucida* (97.27% ANIb; 97.58% ANIm) and *A. salmonicida* subsp. *smithia* (97.21% ANIb; 97.57% ANIm). The top matches were with subsp. *masoucida* (97.27% ANIb; 97.58% ANIm) and subsp. *smithia* (97.21% ANIb; 97.57% ANIm). Since ANI values above 95% denote the same species, these results additionally confirm that SL0n belongs to *A. salmonicida* species.

No plasmids or transposable elements were detected in the SL0n genome. Prophage analysis using PHASTER identified two prophage regions (Table S3), one intact and one questionable. The latter is commonly found in *A. salmonicida* strains and shows high identity to Aeromonas phage phiO18P (Myoviridae family). Notably, neither prophage element harbored virulence-associated or antimicrobial resistance genes.

### 3.3. Virulence Factors

The virulence determinants present in strain SL0n comprise a broad spectrum of factors, including genes involved in adherence, secretion systems, toxin production, and immune evasion, as detailed in Table S4 alongside comparisons with several other Aeromonas species. None of these virulence genes exist within mobile genetic elements in SL0n.

Regarding adherence, the genome contains intact operons encoding several adhesion systems: Flp type IV pili (*flp1-flpL*), Tap type IV pili (*tapB-tapY1*, *tppA-tppF*), mannose-sensitive haemagglutinin (*Msh*) pili (*mshA-mshQ*), and Type I fimbriae (*fimA-fimF*). Also, a complete gene list for polar flagella is present. However, strain SL0n lacks genes for lateral flagella, a characteristic shared by approximately 60% of Aeromonas species [77]. This characteristic is known to lower, but not completely eliminate, its adherence [78]. In contrast, the reference strains *A. salmonicida* subsp. *salmonicida* A449 and *A. salmonicida* subsp. *masoucida* RFAS1 do encode lateral flagellar genes within their chromosomes.

The strain possesses a complete type II secretion system (T2SS) and several genes associated with the type VI secretion system (T6SS). Consistent with its plasmid-free status, SL0n lacks the type III secretion system (T3SS) commonly found among Aeromonas species [79].

A diverse set of toxin-encoding genes is present within the genome, including aerolysin (*aerA*), multiple haemolysins (*ahh1*, *hlyA*, *hly-III*, *TH*), and a complete repertoire of RTX toxins (*rtxA*–*rtxH*). The same diverse set of toxins was found in *A. salmonicida* subsp. *oncorhynchi* A-9, *A. salmonicida* subsp. *pectinolytica* 34 mel, and reference *A. hydrophila* subsp. *hydrophila* ATCC 7966 strains. Regarding immune evasion genes, strain SL0n harbors the *wecC* gene (alone from its operon *wecA–G*), which participates in enterobacterial common antigen (ECA) biosynthesis, and genes associated with an Acinetobacter-like capsule (*fdtA–fdtC*, *rmlA*).

### 3.4. Antimicrobial Resistance Genes

Resistance gene analysis using the Resistance Gene Identifier (RGI) detected 8 antimicrobial resistance (AMR) genes in strain SL0n: 1 perfect match and 7 strict matches. These included *OXA-956* (conferring penicillin resistance), *adeF* (associated with fluoroquinolone and tetracycline resistance), *cph5* (mediating carbapenem resistance), and *FOX-18* (responsible for cephalosporin resistance), as detailed in Table S5. Such resistance determinants are commonly found among Aeromonas strains worldwide.

### 3.5. LD50 Determination and Clinical Picture of Acute Infection

For *A. salmonicida* SL0n, the 4-day median lethal dose (LD50) was determined to be 1.63 × 10^6^ CFU/fish (95% CI: 1.18 × 10^6^–2.23 × 10^6^ CFU) (Figure S1a). Doses below 3.2 × 10^5^ CFU did not cause significant fish mortality, while doses above 6.0 × 10^6^ CFU resulted in 100% mortality among the experimental individuals. The onset of mortality at high doses was observed within 24 h of the experiment (Figure S1b).

Behavioral changes in fish initially manifested as reduced motor activity and a tendency to remain in the water inflow zone or at the bottom of the tank. In later stages, or when exposed to high doses of the pathogen, coordination disorders and chaotic swimming developed. Additionally, individuals with severe skin lesions exhibited “flashing,” characterized by rubbing their bodies against the walls of the tank.

The first signs of disease were observed 18–24 h after injection and manifested as behavioral abnormalities and skin damage. Clinical examination of the fish at the end of the acute experiment (96 h) showed distinct clinical signs depending on the dose of *A. salmonicida* SL0n. At doses up to 6.5 × 10^5^ CFU/fish, local inflammation was observed at the injection site, characterized by distinct borders and light coloration (Figure S2a). At a higher dose (1.5 × 10^6^ CFU/fish), the area of inflammation at the injection site was more extensive and showed signs of tissue necrosis while maintaining the integrity of the skin (Figure S2b). When the dose was increased to 3.0 × 10^6^ CFU, extensive ulcerative lesions were observed on the dorsal part of the body (Figure S2c). Additionally, some fish showed exophthalmos and hemorrhages on the fins and ventral part of the body. At 6.0 × 10^6^ CFU/fish, the dose that resulted in minimal survival, the fish developed extensive and pronounced hemorrhagic dermatitis and ulcers (Figure S2d). Some fish showed areas of tissue loss and exposure of the underlying muscles. In the control group of fish injected with PBS, no inflammation or damage to the external integument was observed on day 4.

Pathological changes in the abdominal organs included: (1) congestive phenomena, such as blood congestion in the liver and spleen; (2) the presence of hemorrhagic foci; (3) the accumulation of serous or hemorrhagic fluid in the abdominal cavity; and (4) gill anemia. Samples collected from the muscle tissue at the injection site and the liver of fish with clinical signs confirmed the presence of *A. salmonicida* within the organism.

### 3.6. Clinical Picture in Prolonged Experiment

**Figure 3:**
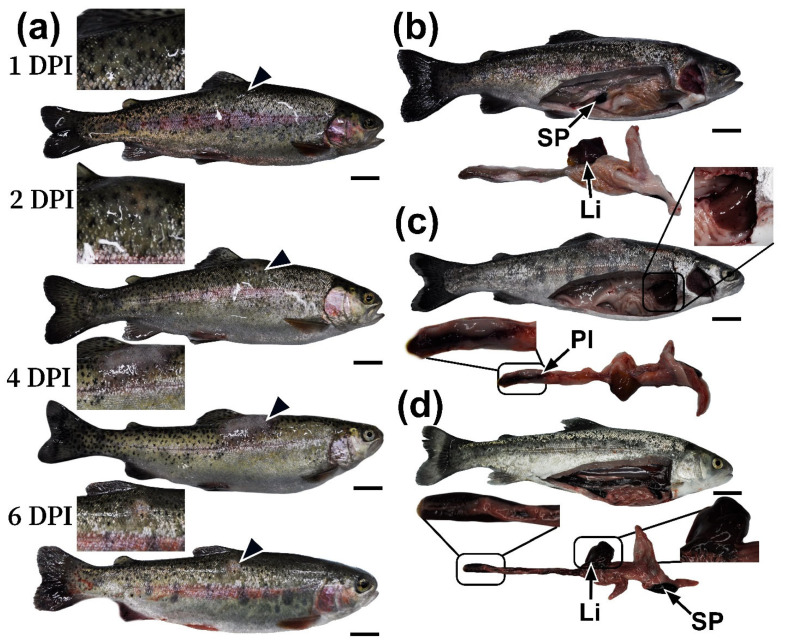
Appearance of rainbow trout at 1–6 DPI injected with *A. salmonicida* SL0n in a prolonged experiment: (**a**) skin lesions (arrowhead) at various periods of the experiment in infected fish; (**b**) condition of internal organs in control fish at 0 DPI; (**c**) condition of internal organs in infected fish at 2 DPI; and (**d**) 4 DPI. Abbreviations: Sp—spleen, Li—liver, PI—posterior intestine. Scale bar 2 cm.

As part of the prolonged experiment, at 1 DPI, the control and experimental groups showed no significant pathological abnormalities beyond for edema and pigmentation changes at the injection site. The condition of the internal organs was normal in both groups (Figure 3a,b). Nevertheless, biological material was collected for hematological, biochemical, and histological studies to investigate the initial stages of pathogenesis.

On the second day (2 DPI), some individuals in the experimental group showed the first pathological changes, namely an increase in the area of redness and edema at the injection site, indicating the initial stage of an inflammatory response. Upon gross examination of the fish, a significant increase in the size of the liver and spleen was observed; these organs were dark in color (Figure 3c), indicating congestive phenomena. The gastrointestinal tract, especially the hindgut, showed focal hemorrhages and inflammatory changes. The gills appeared dark. No visible abnormalities were observed in the kidneys.

On the fourth day (4 DPI), the injection site was characterized by severe damage to the skin, manifested by edema and significant inflammation of the underlying tissue (Figure S4). Autopsy of individuals with obvious clinical signs revealed systemic damage to the abdominal organs. Marked hepatosplenomegaly was observed, with the liver and spleen appearing dark in color and containing multiple areas of necrosis and hemorrhage (Figure 3d). Throughout the intestine, catarrhal or hemorrhagic enteritis was observed, characterized by hyperemia and thinning of the walls, as well as the presence of bloody exudate in the lumen. The gills were dark in color and had diffuse hemorrhages. The trunk kidney was edematous, with isolated petechial hemorrhages (Figure S3).

By the sixth day of the experiment (6 DPI), partial regression of pathological changes was observed in the remaining individuals. Compared to 4 DPI, both skin lesions and gastrointestinal inflammation were less pronounced. However, affected skin areas remained edematous, and the spleen and liver tissues were still congested. Renal edema and severe congestion of the gills persisted. After 6 DPI, no fish deaths were recorded in the experimental group. By 8–10 DPI, all external clinical signs of the disease had resolved. Notably, 13.3% (*n* = 4) of infected individuals remained asymptomatic throughout the observation period.

### 3.7. Bacterial Load

To assess the spread of bacteria in the fish’s body, an analysis of the bacterial load in the internal organs was performed (Table 1). No microorganisms were detected in the tissues of the control group throughout the experiment. In infected individuals at 1 DPI, *A. salmonicida* was detected in the blood (0.02 × 10^3^ CFU/mL) and liver tissue (1.24 × 10^3^ CFU/g). By 2 DPI, the bacterial load reached peak values of 3.24 × 10^5^ CFU/g in liver tissue. By 4 DPI, the number of bacteria in the tissues studied had decreased. At 6 DPI, *A. salmonicida* was detected exclusively in the liver at a concentration of 0.04 × 10^3^ CFU/g.

### 3.8. Hematological Parameters

**Figure 4:**
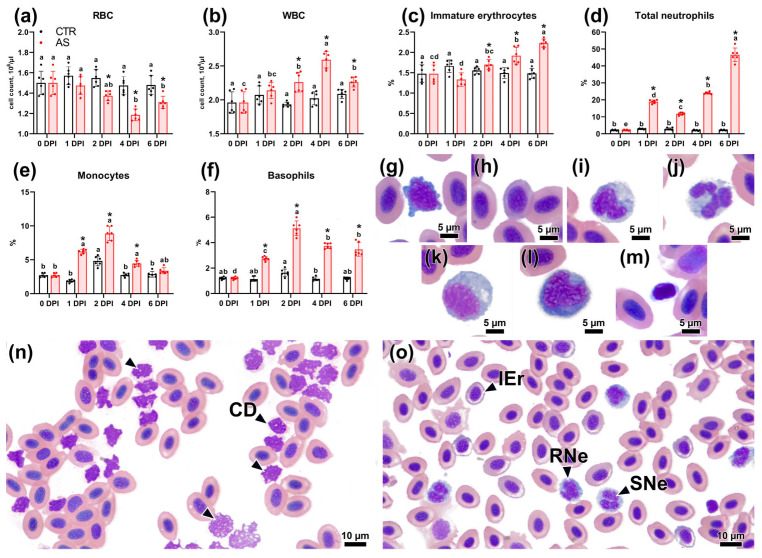
The figure illustrates hematological parameters of rainbow trout from control and infected groups, along with images of specific blood cell types and overall blood pictures at different time points. Panels (**a**–**f**) display hematological parameters. Representative images of blood cells include: (**g**) lymphocyte, (**h**) immature erythrocyte, (**i**) rod-shaped neutrophil, (**j**) segmented neutrophil, (**k**) monocyte, (**l**) basophil, and (**m**) thrombocyte. Panels (**n**) and (**o**) show the overall blood picture at 4 DPI and 6 DPI, respectively. Abbreviations: CD—cell debris; IEr—immature erythrocyte; RNe—rod-shaped neutrophil; SNe—segmented neutrophil. Scale bars are 5 µm for panels (**g**–**m**) and 10 µm for panels (**n**,**o**). Note: Superscript letters (a, b, c, d) denote statistical significance (*p* < 0.05 or *p* < 0.01) within a group across different time points. Asterisks (*) indicate statistical significance between the control (CTR) and experimental (AS) groups at a given time point. The exact *p*-values are provided in Supplementary Table S7.

A study of hematological parameters in infected fish revealed notable changes. The erythrocyte count decreased significantly (*p* < 0.05) compared to the control group, starting at 1 DPI (1.37 × 10^6^ cells/µL) and reaching a minimum at 4 DPI (1.19 × 10^6^ cells/µL) (Figure 4a). Concurrently, the immature erythrocyte initially decreased slightly at 1 DPI and then gradually increased (Figure 4c). The level of immature erythrocytes at 6 DPI differed significantly from both the corresponding control group and the experimental fish at other time points (*p* < 0.01). The white blood cell count significantly increased (*p* < 0.01) in the experimental group as early as 2 DPI, with the highest level observed at 4 DPI, reaching 2.59 × 10^4^ cells/µL (Figure 4b). Examination of blood smears from experimental fish on days 2 and 4 post-infection revealed numerous cell fragments (cell ghosts) among the erythrocytes. These fragments were basophilic and irregularly shaped, indicating they formed via hemolysis (Figure 4n). The list of abbreviations for hematological and histological parameters is provided in Table S12.

The neutrophil count exhibited increased linearly, significantly exceeding values in the control group as early as 1 DPI (Figure 4d). The highest neutrophil count was observed at 6 DPI, significantly exceeding (*p* < 0.01) both the control group and other experimental time points. At 6 DPI, blood smears from infected fish contained up to 5–8 neutrophils per field of view (Figure 4o). Concurrently, the count of rod-shaped forms exceeded segmented forms only at 1 DPI (RNe:SNe = 13.11:5.83%), whereas at 6 DPI, segmented forms were significantly more frequent (RNe:SNe = 14.55:32.05%) (Table S6). Experimental fish exhibited a significant increase in monocyte levels, peaking at 2 DPI (8.87%). Subsequently, their percentage decreased at 4 and 6 DPI, yet it remained higher than in the control group, which also displayed a transient, significant increase in monocytes at 2 DPI (*p* < 0.05) (Figure 4e). The basophil level in the experimental group showed a similar pattern: a marked increase at 1 and 2 DPI, followed by a decrease at 4 and 6 DPI (Figure 4f). These changes were statistically significant compared to the control group at all specified points (*p* < 0.01). Thrombocyte levels decreased significantly in the experimental group at 1 DPI (to 0.66%), followed by recovery by 4 and 6 DPI (Table S6).

### 3.9. Blood Biochemistry

**Figure 5:**
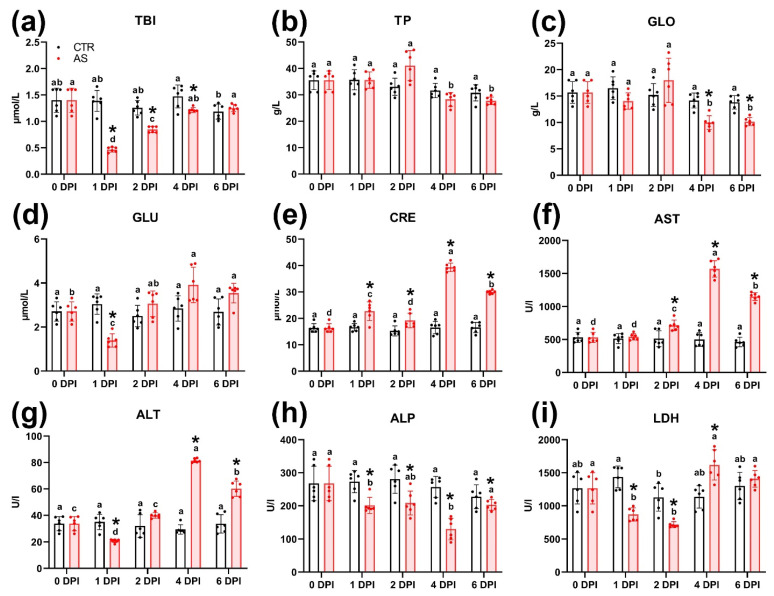
Biochemical parameters of rainbow trout blood serum in the control and infected groups at various time points (**a**–**i**). Note: Superscript letters (a, b, c, d) denote statistical significance (*p* < 0.05 or *p* < 0.01) within a group across different time points. Asterisks (*) indicate statistical significance between the control (CTR) and experimental (AS) groups at a given time point. The exact *p*-values are provided in Supplementary Table S7.

Most biochemical parameters in the blood of infected rainbow trout changed over the course of the experiment. Total bilirubin concentration in infected individuals was significantly lower (*p* < 0.01) than in the control group from 1 to 4 DPI, subsequently recovering to control values by 6 DPI (Figure 5a). Notably, the control group also showed a significant decrease (*p* < 0.05) in total bilirubin by 6 DPI compared to 4 DPI (Figure 5a). No significant differences in total protein content were observed between the control and infected groups at any time point (Figure 5b). However, within the infected group, the total protein level peaked at 41.08 g/L on 2 DPI, a value significantly (*p* < 0.01) higher than levels at 4 and 6 DPI. The dynamics of albumin and globulin fraction concentrations were similar (Table S8); however, significant differences (*p* < 0.01) were found in globulin concentrations between the control and infected groups at 4 and 6 DPI (Figure 5c).

At 1 DPI, blood glucose in infected fish dropped significantly (*p* < 0.05), a level lower than both the control group at that time and the infected group at later time points (Figure 5d). Glucose concentration in the infected group peaked at 4 DPI, but this level was not significantly different from the control group. Urea concentration also showed a significant decrease at 1 DPI (Table S8), after which it recovered to control values by 2–6 DPI. Creatinine concentration in the infected group differed significantly (*p* < 0.05 and *p* < 0.01) from control values at all time points, peaking sharply at 4 DPI (39.35 µmol/L) before decreasing at 6 DPI (29.98 µmol/L) (Figure 5e).

The activity of aminotransferases (AST and ALT) significantly increased (*p* < 0.01) at 4 and 6 DPI, markedly exceeding levels both the control group and the infected group at earlier time points (Figure 5f,g). Alkaline phosphatase (ALP) and lactate dehydrogenase (LDH) activity significantly decreased (*p* < 0.01) in infected individuals at 1 and 2 DPI (Figure 5h,i). This decrease was significant compared to the control group at the corresponding time points. For ALP, this low activity persisted until 4 DPI. LDH activity then increased significantly at 4 DPI (*p* < 0.05), exceeding the control value.

### 3.10. Histology of Muscle Tissue at the Injection Site

**Figure 6:**
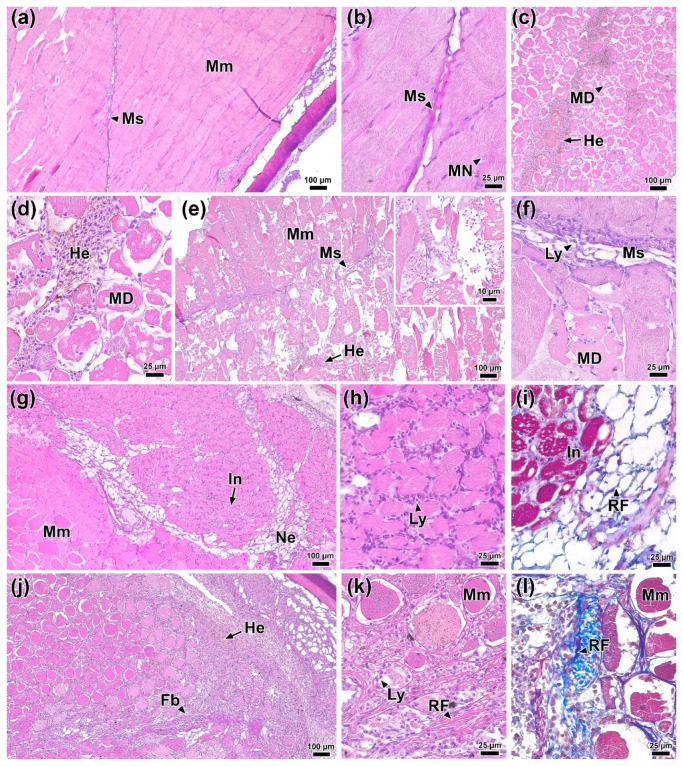
Histological sections of the dorsal musculature of rainbow trout before (**a**,**b**) 0 DPI and after infection: (**c**,**d**) 1 DPI, (**e**,**f**) 2 DPI, (**g**–**i**) 4 DPI, and (**j**–**l**) 6 DPI. Abbreviations: MS—myoseptum, Mm—myomer, MN—myomer nucleus, MD—myomer degradation, He—hemorrhage, Ly—lymphocyte, Ne—necrosis, In—infiltration, RF—reticular fiber, Fb—fibrosis. H & E staining (**a**–**h**,**j**,**k**) and MT staining (**i**,**l**), scale bar 100 (**a**,**e**,**g**,**j**), 25 (**b**–**d**,**f**,**h**,**i**,**k**,**l**) and 10 µm.

Histological sections of rainbow trout muscle tissue prior to infection (0 DPI) clearly showed individual myocytes organized into myomers and separated by myosepta (Figure 6a). Small elongated nuclei were visible within the myocytes (Figure 6b).

One day after infection (1 DPI), foci of hemorrhage and edema, along with pronounced degradation of individual muscle fibers, were present at the injection site and in the surrounding area (Figure 6c). However, at this time, no signs of immune cell infiltration were evident (Figure 6d). In the control group injected with PBS, similar structural changes in muscle tissue were observed (Figure S5a). By 2 DPI, the area of muscle tissue damage had increased (Figure 6e). Disruption of myocyte structure included the condensation of myofibrils and their separation from the sarcolemma. Additionally, in the myoseptum area, near the affected regions, the presence of individual immunocompetent cells, primarily lymphocytes, was noted (Figure 6f).

At 4 DPI, lesions similar to those observed at 2 DPI were present in the muscle tissue near the injection site. Furthermore, necrotic areas were identified surrounding regions of tissue exhibiting marked myocyte atrophy and immune cell infiltration (Figure 6g,h). These areas were often located adjacent to intact tissue. These necrotic areas lacked muscle fibers and contained a network of collagen matrix (Figure 6i). The control group at 4 DPI showed no pronounced regressive changes; however, areas of muscle tissue contained a significant amount of collagen fibers and blood cells (Figure S5b)

By 6 DPI, regression of lesions was observed. Areas of damaged muscle fibers were replaced by connective tissue (fibrosis) characterized by a high content of reticular fibers, among which various blood cell elements were located (Figure 6j–l). There were no significant foci of hemorrhage or infiltration of muscle tissue in the studied individuals at this time point.

### 3.11. Histology of the Hindgut

**Figure 7:**
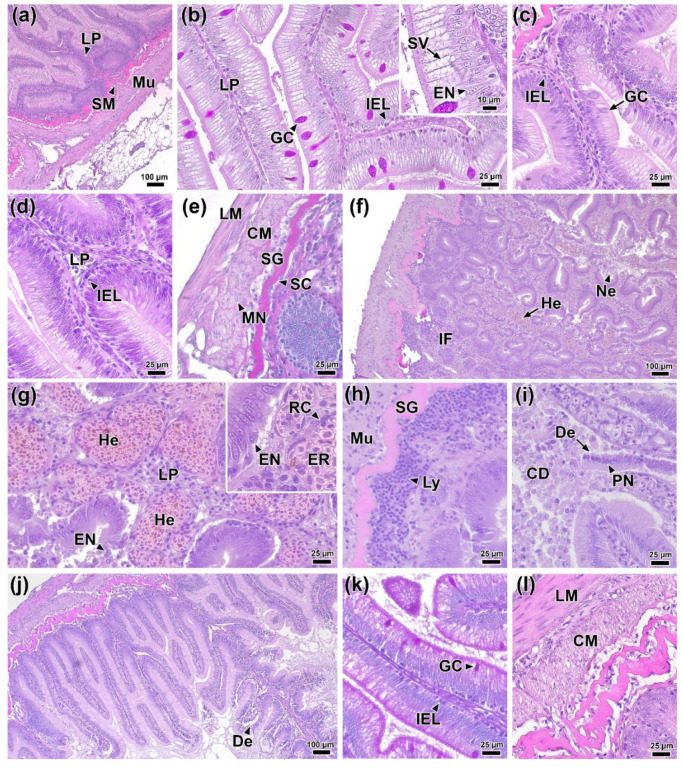
Histological sections of the posterior intestine of rainbow trout before (**a**,**b**) 0 DPI and after infection: (**c**) 1 DPI, (**d**,**e**) 2 DPI, (**f**–**i**) 4 DPI, and (**j**–**l**) 6 DPI. Abbreviations: LP—lamina propria, SM—submucosa, Mu—muscularis, GC—goblet cell, EN—epithelial cell nucleus, SV—supranuclear vacuoles, IEL—intraepithelial lymphocytes, LM—longitudinal muscle layer, CM—circular muscle layer, SG—stratum granulosum, SC—stratum compactum, MN—myocyte nucleus, IF—inflammation focus, He—hemorrhage, Ne—necrosis, RC—reticular fiber, Er—erythrocyte, Ly—lymphocytes, CD—cell debris, De—desquamation, PN—pyknotic nuclei. H & E staining (**a**,**c**,**d**,**f**–**j**,**l**) and PAS staining (**b**,**e**,**k**), scale bar 100 (**a**,**f**,**j**), 25 (**b**–**e**,**g**–**i**,**k**,**l**) and 10 µm.

The distal intestine was selected for histopathological examination due to macroscopic pathological changes detected there during both the acute and prolonged phases of the experiment. The normal histological structure of the posterior intestine comprised: a folded mucous layer; lamina propria, consisting of connective tissue with vascular inclusions and individual immunocompetent cells; a submucosa of compact and granular layers; and a muscularis with two layers of smooth muscle—the inner longitudinal and outer transverse (Figure 7a). The epithelium of the mucosa consisted of columnar epithelial cells with pronounced apical vacuolization (supranuclear vacuoles) and goblet cells (Figure 7b).

**Figure 8:**
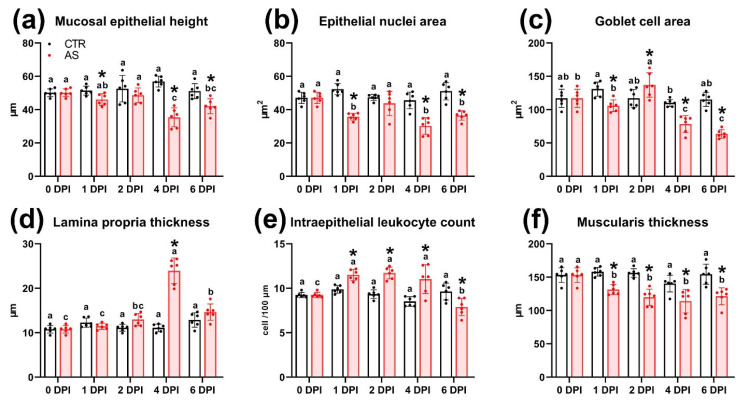
Histomorphometric parameters of tissue elements in the posterior intestine of rainbow trout in the control and infected groups (**a**–**f**). Note: Superscript letters (a, b, c) denote statistical significance (*p* < 0.05 or *p* < 0.01) within a group across different time points. Asterisks (*) indicate statistical significance between the control (CTR) and experimental (AS) groups at a given time point. The exact *p*-values are provided in Supplementary Table S7.

At 1 DPI, no significant morphological abnormalities showed in intestinal tissues. However, morphometric analysis revealed a significant decrease in the area of epithelial cell nuclei, an increase in the number of intraepithelial lymphocytes, and a thickening of the muscle layer compared to the control group (Figure 7c; Figure 8b,e,f; Table S9). In addition, a statistically significant decrease (*p* < 0.01) in the average area of goblet cells to 105.8 µm^2^ was observed in the experimental group, whereas this value in the control group was 157.9 µm^2^ (Figure 8e).

By 2 DPI, some areas of the longitudinal muscle layer showed marked edema, evidenced by a decrease in tissue chromophilia and structural integrity (Figure 7e). Additionally, changes were detected in the mucous membrane, manifested as a significant increase (*p* < 0.01) in the number of intraepithelial leukocytes (Figure 7d) and a decrease in the thickness of the muscular layer. The hindgut tissues of control fish showed no significant morphological abnormalities throughout the experimental period.

At 4 DPI, the most pronounced histopathological changes exhibited in the intestines of experimental fish. Extensive hemorrhages and foci of necrosis of the mucosal epithelium appeared throughout the examined sections (Figure 7f). In the lamina propria of the mucous membrane, capillary dilatation and hyperemia were observed, accompanied by reticular tissue hypertrophy (Figure 7g). During this period, the thickness of the lamina propria in the infected group reached its maximum value for the entire observation period (23.9 µm; *p* < 0.01) (Figure 8d). Large clusters of immunocompetent cells were present near the submucosa (Figure 7h), and the number of intraepithelial lymphocytes was 11.03 cells/100 µm, significantly exceeding (*p* < 0.01) the control group values (Figure 8e). In areas of mucosal necrosis, desquamation of epithelial cells with pyknotic nuclei and abundant cell debris in the intestinal lumen were observed (Figure 7i). Morphometric measurements confirmed these observations, showing that at 4 DPI, the height and area of the epithelial nucleus were the smallest (*p* < 0.01) of the entire experiment in the infected group (Figure 8a,b).

At the end of the experiment (6 DPI), areas of necrosis and desquamation of the mucosal epithelium persisted in the intestinal tissues of infected fish, but their prevalence was significantly lower (Figure 7j). No major hemorrhages or clusters of immunocompetent cells were detected. Additionally, the area of goblet cells and the number of intraepithelial lymphocytes decreased significantly (*p* < 0.05 and *p* < 0.01, respectively). In the muscle layer, histological abnormalities similar to those at 2 DPI were present, notably, myocyte edema (Figure 7i). Morphometric data revealed that the muscle layer in the infected group was significantly thinner (*p* < 0.01) than in the control (Figure 8f).

### 3.12. Liver Histology

**Figure 9:**
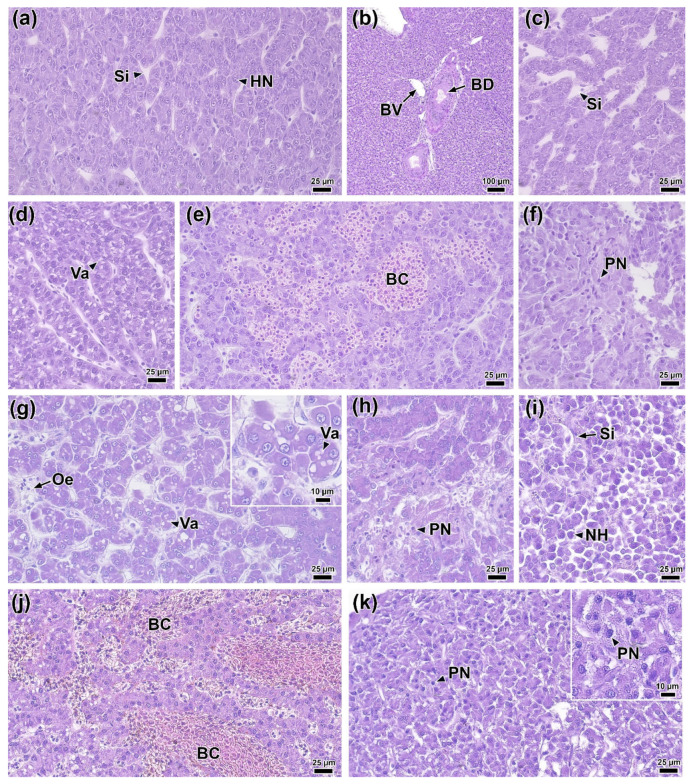
Histological sections of rainbow trout liver before infection (**a**,**b**) 0 DPI and after infection: (**c**,**d**) 1 DPI, (**e**,**f**) 2 DPI, (**g**–**i**) 4 DPI (**j**,**k**) and 6 DPI. Abbreviations: HN—hepatocyte nucleus, Si—sinusoidal capillary, BV—blood vessel, BD—bile duct, Va—vacuolization, BC—blood stasis, PN—pyknotic nucleus, Oe—edema, NH—necrotic hepatocyte. H & E staining, scale bar 100 (**b**), 25 (**a**,**c**–**k**) and 10 μm.

The histology of rainbow trout liver tissue at the start of the experiment (0 DPI) was normal for this species of fish. Hepatocytes with dense cytoplasm and round basophilic nuclei were organized into anastomotic cords separated by sinusoidal capillaries (Figure 9a). The liver tissue also contained blood vessels and bile ducts (Figure 9b).

**Figure 10:**
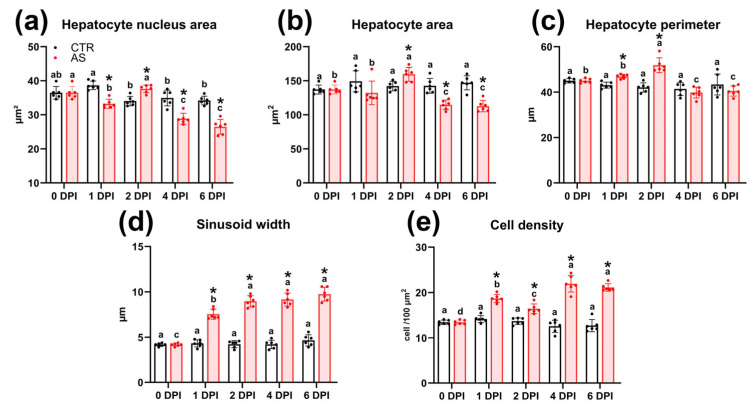
Histomorphometric parameters of liver tissue elements in rainbow trout in the control and infected groups (**a**–**e**). Note: Superscript letters (a, b, c, d) denote statistical significance (*p* < 0.05 or *p* < 0.01) within a group across different time points. Asterisks (*) indicate statistical significance between the control (CTR) and experimental (AS) groups at a given time point. The exact *p*-values are provided in Supplementary Table S7.

At 1 DPI, the livers of infected fish showed multifocal dilatation of sinusoidal capillaries (Figure 9c). Morphometric analysis confirmed that their average width significantly (*p* < 0.01) exceeded the control values (Figure 10d; Table S10). Some sections showed focal vacuolization of hepatocytes, which contained vacuoles of variable size with irregular borders (Figure 9d). During the same period, the area of hepatocyte nuclei decreased significantly (33.2 μm^2^; *p* < 0.01), while cell density increased (18.7 cells/100 μm^2^; *p* < 0.01) compared to the control (Figure 10a,e).

By 2 DPI, the dilation of sinusoidal capillaries had become diffuse; their lumen was enlarged and filled with erythrocytes (Figure 9e). Additionally, foci of necrosis were evident, with hepatocytes exhibiting pyknotic nuclei and indistinct cell boundaries (Figure 9f). Simultaneously, the area of hepatocytes at 2 DPI significantly (*p* < 0.05) increased compared to both the control and 1 DPI experimental groups (Figure 10b).

At 4 DPI, histopathological changes in the liver of infected fish reached their maximum severity. In all samples, foci of edema and vacuolization of hepatocytes were recorded (Figure 9g). These foci were characterized by expanded intercellular spaces and hepatocytes exhibiting moderate nuclear pleomorphism and rounded cytoplasmic vacuoles. Necrotic changes became more widespread compared to 2 DPI and presented as two morphological types. The first type consisted of small foci with blurred boundaries, containing hepatocytes with signs of pyknosis and karyorrhexis, and abundant cellular debris (Figure 9h). The second type included large foci of necrosis with morphological signs of apoptosis, such as cytoplasmic condensation and nuclear pyknosis, in the absence of pronounced leukocyte infiltration (Figure 9i). Morphometric analysis showed a statistically significant (*p* < 0.01) decrease in the area of the nucleus and cytoplasm of hepatocytes (Figure 10a,b), and an increase in the lumen of sinusoids and cell density compared to the control group (Figure 10d,e).

By 6 DPI, the overall intensity of pathological changes had decreased. Nevertheless, congestive phenomena persisted in the liver including erythrocyte stasis in sinusoidal capillaries (Figure 9j) and extensive areas of hepatocyte necrosis without signs of infiltration (Figure 9k). Several morphometric parameters remained unchanged from 4 DPI levels, maintaining statistically significant differences from the control (Figure 10).

### 3.13. Histology of the Trunk Kidney

**Figure 11:**
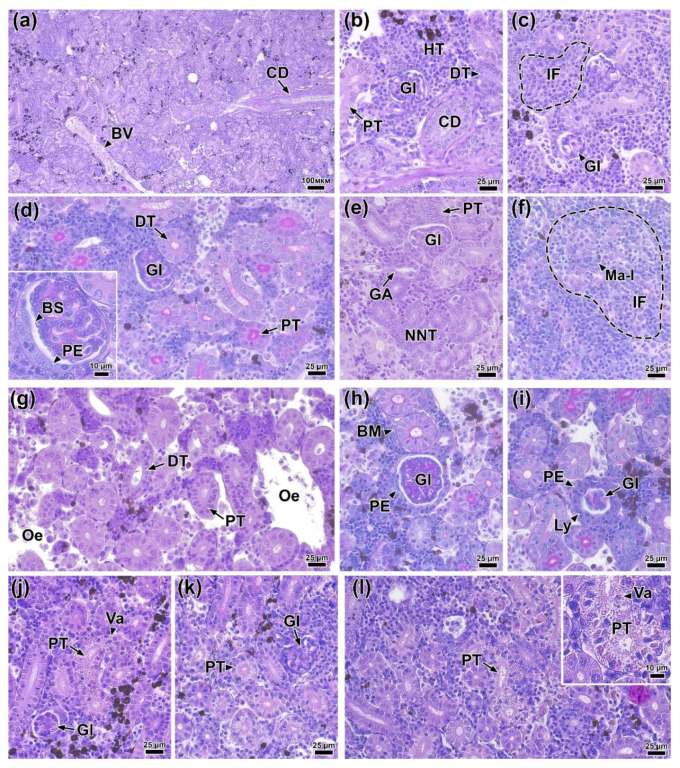
Histological sections of the trunk kidney of rainbow trout before (**a**,**b**) 0 DPI and after infection: (**c**) 1 DPI, (**d**–**f**) 2 DPI, (**g**–**j**) 4 DPI and (**k**,**l**) 6 DPI. Abbreviations: BV—blood vessel, CD—collecting duct, HT—hematopoietic tissue, DT—distal renal tubule, PT—proximal renal tubule, GL—glomerulus, BS—Bowman’s space, PE—parietal epithelium, GA—glomerular arteriole, NNT—neonephrogenic tubules, Ma-l—macrophage-like cell, IF—inflammation, Oe—edema, BM—basement membrane, Ly—lymphocyte, Va—vacuolization. H & E staining (**a**–**c**,**e**,**g**,**j**–**l**) and PAS staining (**d**,**f**,**h**,**i**), scale bar 100 (**a**), 25 (**b**–**l**) and 10 μm.

At 0 DPI (before the experiment), the following morphofunctional elements were clearly distinguishable in the trunk kidney tissue: the renal corpuscle (glomerulus) located in Bowman’s capsule; the proximal and distal sections of the nephron, differing in the thickness of the cuboidal epithelium and the development of the brush border; hematopoietic tissue and blood vessels; and collecting ducts with an enlarged basal membrane of the epithelium (Figure 11a,b).

On the first day (1 DPI), infected fish showed small foci of inflammation in the hematopoietic tissue, characterized by the presence of cells with large nuclei and amphiphilic cytoplasm (Figure 11c). In addition, some glomeruli showed structural disruption, evidenced by a decrease in the area of Bowman’s space and hypercellularity of the glomerulus. Morphometric measurements of kidney tissue elements did not reveal statistically significant differences from the control (Table S11). The group of fish injected with PBS did not show any pathological abnormalities in the kidney throughout the entire experiment.

**Figure 12:**
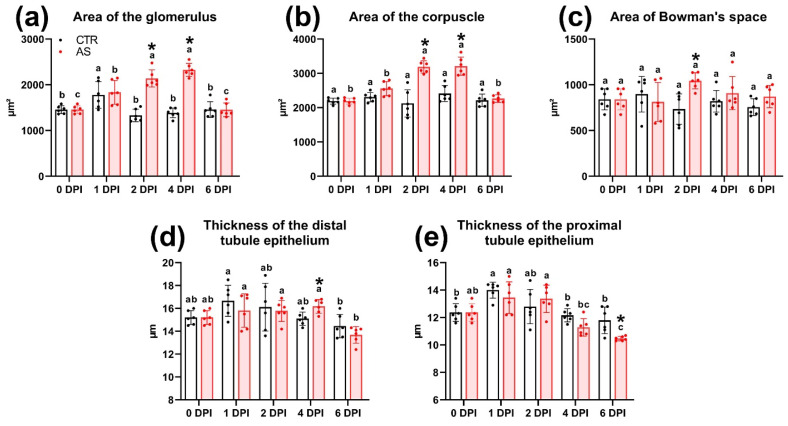
Histomorphometric parameters of tissue elements of the trunk kidney of rainbow trout in the control and infected groups (**a**–**e**). Note: Superscript letters (a, b, c) denote statistical significance (*p* < 0.05 or *p* < 0.01) within a group across different time points. Asterisks (*) indicate statistical significance between the control (CTR) and experimental (AS) groups at a given time point. The exact *p*-values are provided in Supplementary Table S7.

On the second day (2 DPI), the severity of morphological abnormalities in the infected fish was significantly higher. Most glomeruli showed signs of hypercellularity, likely associated with mesangial cell proliferation. Additionally, the parietal epithelium was significantly hypertrophied (Figure 11d). A large number of neonephrogenic tubules with pronounced basophilic staining and a narrow lumen were observed throughout the section (Figure 11e). The prevalence and severity of inflammatory foci were higher than on 1 DPI. Cells with large irregular nuclei and PAS-positive cytoplasm (macrophage-like cells) were also found in these areas (Figure 11f). Measurements of tissue elements showed a significant increase (*p* < 0.01) in the area of the corpuscle, glomerulus, and Bowman’s space compared to the control (Figure 12a–c). However, no significant morphological changes in the renal tubules were detected.

At 4 DPI, kidney tissue from infected fish exhibited pronounced edema, characterized by cell-free areas containing a small number of cellular elements and cell debris (Figure 11g). Glomerular abnormalities were similar to those at 2 DPI and included hypertrophy of the parietal epithelium and glomerular hypercellularity. Additionally, renal corpuscles with severely disrupted morphology were observed in the tissue, exhibiting a significantly enlarged parietal epithelium infiltrated by scattered leukocytes and deformed capillary loops (Figure 11i). Morphometric analysis showed a significantly larger (*p* < 0.01) area of the glomerulus and corpuscle in the experimental group compared to the control and to the injected group at 1 and 6 DPI (Figure 12a,b). In addition, the distal tubule epithelium became significantly thicker (*p* < 0.05), while the proximal tubule epithelium became thinner (Figure 12d,e).

At 6 DPI, the kidney tissue of the experimental group showed decreased edema severity, while glomerular structural damage persisted and was morphologically similar to that observed at 2 and 4 DPI (Figure 11j,k). The epithelium of some proximal tubules showed signs of atrophy and vacuolization. The rounded vacuoles, varied in size, were eosinophilic, which may indicate hyaline degeneration (Figure 11j,l). The cuboidal epithelium of both the proximal and distal tubules was significantly thinner (*p* < 0.05) compared to the control (Figure 12d,e). These values reached their minimum at 6 DPI and were significantly lower (*p* < 0.01) than at other time points.

### 3.14. Histology of the Spleen

**Figure 13:**
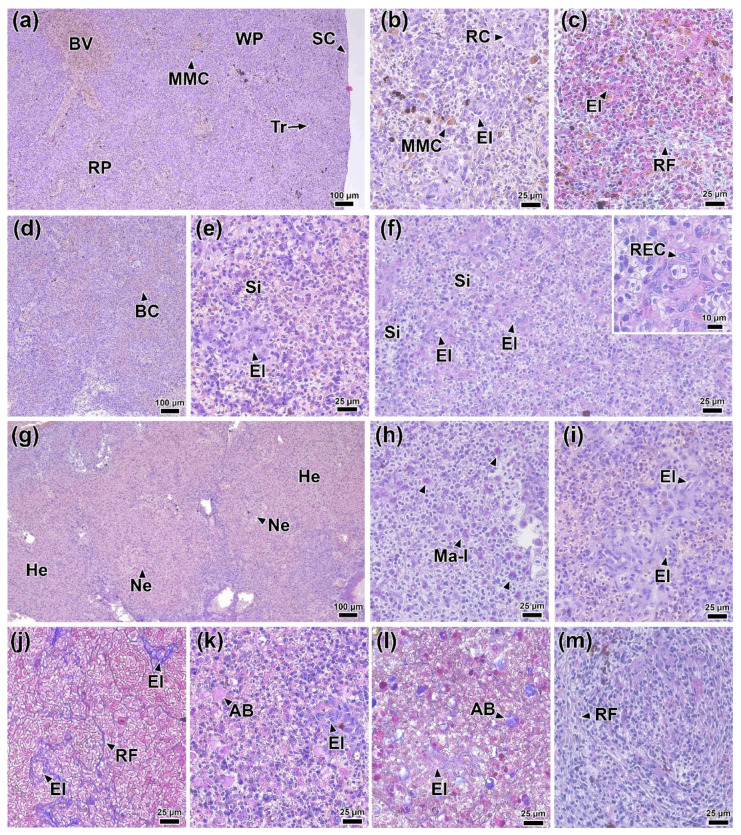
Histological sections of rainbow trout spleen before at (**a**–**c**) 0 DPI and after infection: 1 (**d**) DPI, (**e**–**f**) 2 DPI, (**g**–**j**) 4 DPI, and (**k**–**m**) 6 DPI. Abbreviations: BV—blood vessel, RP—red pulp, WP—white pulp, MMC—melanomacrophage centers, Tr—trabecula, SC—spleen capsule, El—ellipsoid, RC—reticular cell, RF—reticular fiber, BC—blood stasis, REC—reticuloendothelial cell, He—hemorrhages, Ma-l—macrophage-like cell, AB—apathetic bodies. H&E staining (**a**,**b**,**d**,**e**,**g**,**i**,**k**), PAS staining (**f**,**h**,**m**), and MT staining (**c**,**j**,**i**); scale bar 100 (**a**,**d**,**g**), 25 (**b**,**e**,**f**,**h**–**m**), and 10 µm.

In the spleen of fish at 0 DPI, differentiation of the parenchyma into red and white pulp was indistinct. The stroma of the organ consisted of a connective tissue capsule with individual trabeculae extending from it, transitioning into a network of reticular tissue (Figure 13a,c). Large blood vessels, ellipsoids, capillaries containing erythrocytes, and small melanomacrophage centers with characteristic yellow pigmentation were present in the parenchyma (Figure 13b).

On day 1 post-infection, signs of hyperemia were observed in the spleen: the red pulp sinuses were dilated and filled with erythrocytes. Concurrently, atrophy of discrete areas of white pulp was observed (Figure 13d). No necrotic changes or significant abnormalities in the white pulp tissue were detected. No significant morphological abnormalities were found in control fish at 1 DPI.

At 2 DPI, capillary dilation and hyperemia persisted at the same level. In addition, hypertrophy of the reticular endothelial cells of the spleen ellipsoids was observed (Figure 13e). These cells were characterized by moderately basophilic and PAS-positive cytoplasm, as well as oval or bean-shaped nuclei (Figure 13f).

At 4 DPI, the most prominent pathological changes were observed in the spleen of infected fish. Extensive hemorrhages with foci of necrosis containing abundant cellular debris were present throughout the section (Figure 13g). Large cells with areas of PAS-positive cytoplasm, morphologically similar to macrophages, were also found in the necrotic areas (Figure 13h). The structure of the ellipsoids was significantly altered. Key changes included marked hypertrophy of most reticular endothelial cells, deformation of their nuclei, increased reticular fiber density, and significant enlargement of the ellipsoid lumen (Figure 13i,j).

At 6 DPI, the severity of hemorrhagic manifestations and capillary hyperemia decreased significantly. Multiple rounded, homogeneous eosinophilic inclusions, identified as apoptotic bodies/cells, were present throughout the organ (Figure 13k,l). Hypertrophy of reticular endothelial cells of ellipsoids was less pronounced compared to 4 DPI (Figure 13k). In addition, some areas of the tissue contained connective tissue nodules consisting of reticular fibers (Figure 13m).

## 4. Discussion

### 4.1. Strain A. salmonicida SL0n

The morphological, physiological, and biochemical properties of the studied *A. salmonicida* SL0n strain generally align with the species description in Bergey’s Manual [46]. Consistent with previous reports for this species, the isolate is non-motile, catalase- and oxidase-positive, and ferments glucose to produce acid and gas [18,80]. Its ability to grow in a medium containing up to 2% NaCl is also typical [37,81]. While the lack of motility was previously considered a distinctive feature of *A. salmonicida*, recent studies have demonstrated the existence of motile and highly virulent strains of this species [8,82]. An atypical characteristic of the described strain is its ability to grow at 37 °C [80]. Although mesophilic *A. salmonicida* isolates were not previously considered pathogenic, this view has been challenged. For instance, strains AB001 [8] and SRW-OG1 [81], which were isolated from diseased fish exhibiting typical signs of bacterial infection, were also able to grow at high temperatures.

The studied *A. salmonicida* SL0n isolate demonstrated resistance to several antimicrobial drugs. Its resistance to bacitracin is typical for Gram-negative bacteria and has been previously reported [81]. Resistance to penicillins in many Aeromonas isolates is attributed to the production of chromosomal β-lactamases, as has been previously documented [83]. Furthermore, the strain exhibited resistance to cephalosporins (cefixime and ceftriaxone), which aligns with data from Bakiyev et al. [8], although other Aeromonas strains remain sensitive to these antibiotics [84]. Notably, this strain displayed sensitivity to ciprofloxacin and gentamicin, a finding corroborated by other authors [8,81,85], suggesting the effectiveness of aminoglycosides and fluoroquinolones against *A. salmonicida*. The intermediate or low inhibition zones observed for chloramphenicol and azithromycin may indicate an initial stage of developing resistance to these drugs.

Whole-genome taxonomic assessment indicates that the *A. salmonicida* SL0n strain represents a new species within the *Aeromonas* genus. Its dDDH values (75.0–78.3%) and ANI metrics (96.99–97.59%) further imply that SL0n is a novel subspecies, although the value and necessity of subspecies delineation remain under discussion within the community [86,87,88]. Although plasmids and transposons rank among the most impacting mobile genetic elements in *Aeromonas*, SL0n lacks both [89]. Nevertheless, it possesses a variety of antimicrobial-resistance and virulence-associated genes, displaying a moderate pathogenic potential. Several of these resistance determinants target antibiotics frequently employed in Russian and global aquaculture, underscoring the strain’s potential significance for fish health. The role of these virulence factors in infection dynamics is considered further below. Finally, SL0n harbors two prophages, one of which is intact. This intact prophage could potentially be induced and explored as a therapeutic agent against this strain [90].

The phenotypic characteristics identified in strain SL0n may be related to its adaptation to the specific environmental conditions of aquaculture (RAS). This finding aligns with the previously described phenotypic diversity of *A. salmonicida* isolates from different regions of the world [18].

### 4.2. Acute Experiment and Virulence of Strain A. salmonicida SL0n

Determination of the median lethal dose (LD50) is a standard method for assessing the virulence of pathogenic microbial isolates. *A. salmonicida* is characterized by a wide range of values for this indicator. Thus, Daly et al. [40] found that the LD_50_ for brook trout (*Salvelinus fontinalis*) when administered intraperitoneally was <10 CFU/individual. According to Izumikawa et al. [91], for dark rockfish (*Sebastes melanops*) via intramuscular infection, the LD_50_ was 1.1 CFU/fish. Strains with such low LD_50_ values are considered highly virulent. Conversely, for other isolates, the LD_50_ can reach significantly higher values. For example, for strain RFAS-1 in dark rockfish, this indicator was 1.5 × 10^6.4^ CFU/fish for intramuscular and 1.5 × 10^5.3^ CFU/fish for intraperitoneal administration [37]. LD_50_ values also vary depending on the host species: for golden crucian carp (*Carassius carassius*), LD_50_/7d was 2.06 × 10^7^ CFU/fish [61], and for common carp (*Cyprinus carpio*), LD_50_/4d was 1.5 × 10^7^ CFU/fish [62]. For comparison, according to Mittal et al. [92], *A. hydrophila* strains with an LD_50_ value around 10^5^ CFU are considered virulent.

In this study, the LD_50_ value for strain SL0n was 1.63 × 10^6^ CFU/fish. It is known that this indicator varies significantly among representatives of the genus *Aeromonas* (*A. veroni*, *A. hydrophila*, *A. salmonicida*, etc.) [38,39]. The lethality caused by the pathogen depends on its virulence factors, the host fish species, and the route of inoculation [37,38,62]. Thus, strain virulence assessment should be based on a comprehensive analysis including the pathological changes it causes, its LD50, and its virulence factor profile.

The pathological changes observed during the induction of acute infection with high doses of the SL0n strain (>6.0 × 10^6^ CFU/fish) were predominantly systemic and generalized, typical of septicemia. Muscle tissue damage, manifested by extensive necrosis and observed at maximum inoculum doses, indicates the expression of toxins (aerolysin, hemolysin, enterotoxin) and an active secretory system (T2SS) in the studied strain [93]. The presence of skin lesions confirms the strain’s ability to successfully replicate in muscle tissue, leading to acute inflammation and necrosis. This dissemination leads to disruption of skin integrity and the formation of characteristic ulcers [32]. The identified pathological changes are consistent with previously described data in the literature and are typical of virulent strains of *A. salmonicida* and other Aeromonas species [10,29,94]. Administration of doses close to the LD_50_ induced the development of an acute form of the disease; however, some individuals exhibited no clinical signs. These observations are consistent with data indicating that infected individuals do not always exhibit clinical signs of furunculosis [34].

Based on these findings, we classify strain SL0n as moderately virulent. Although it causes pathological disorders characteristic of *A. salmonicida* infection, it lacks the full complement of virulence factors (e.g., T3SS, GCAT, A-layer) common to typical *A. salmonicida* ssp. *salmonicida* strains [25]. This moderate virulence was advantageous for our study, as it permitted a detailed examination of pathogenesis. Such an analysis would be unfeasible with highly virulent strains, which typically either cause acute mortality within 24–48 h or establish a chronic infection [34].

### 4.3. First Stage of Pathogenesis (1–2 DPI)

In a prolonged experiment using a dose of 75% of the LD50, clinical signs and pathological changes were minimal during the first two days post-infection (1–2 DPI). However, the organism’s initial response manifested as changes in blood cell composition and blood biochemical parameters. Specifically, a significant increase in the number of neutrophils, monocytes, and basophils indicates the development of a nonspecific immune response aimed at eliminating the pathogen [95], which is typical for infectious diseases [96,97,98]. An increase in the level of band neutrophils serves as an indicator of the initial phase of the body’s response to an infectious agent. This is because these cells have limited functionality [99]; their subsequent differentiation into segmented forms provides a functional reserve for immune defense. Notably, as early as 1 DPI, a significant increase in the total number of leukocytes was observed, indicating absolute leukocytosis.

Bacterial load measurements confirmed a systemic infection, with levels peaking in the blood, liver, and muscle tissue at 2 DPI. This pattern of internal organ colonization is typical for bacteria of the genus *Aeromonas* [14,100] and suggests the likely overcome bodily barriers to reach the systemic bloodstream and subsequently colonize target organs. The presence of genes encoding polar flagella and type I pili in SL0n confirms the strain’s ability to penetrate mucosal barriers and attach to cells. Organ invasion is usually accompanied by damage resulting from the pathogen’s cytotoxic action.

At this stage of pathogenesis, biochemical parameters showed a marked decrease in the concentrations of total bilirubin and glucose, as well as in the activity of ALP and LDH. The decrease in glucose concentration is likely associated with an altered basal metabolism used to overcome disease-induced stress [44]. The immune response to pathogen invasion is energy-intensive, which leads to increased glucose utilization. A similar phenomenon was previously observed in pacu (*Piaractus mesopotamicus*) infected with *A. hydrophila* [101]. Conversely, a significant increase in the concentration of this substrate was observed at 1 DPI in Nile tilapia (*Oreochromis niloticus*) [102]. This suggests that the dynamics of glucose concentration during infection depend on many factors, the most significant likely being the physiological status of the fish. Hypobilirubinemia, most pronounced at 1 DPI, is presumably due to impaired erythrocyte hemolysis and/or bilirubin transport in the spleen [103]. The splenic hyperemia detected during histological examination confirms this hypothesis. Similar biochemical changes were previously described by Řehulka [104] in rainbow trout during the natural course of ulcerative dermatitis caused by Aeromonas.

ALP plays a key role in calcification and lipid transport in the intestine [105]. A sharp decrease in its blood activity at 1–2 DPI may indicate suppression of the liver’s synthetic function. However, no significant damage to hepatocytes was observed at the cellular level at 1 and 2 DPI. This suggests that the decrease in ALP activity may be a consequence of damage to erythrocyte membranes and their hemolysis, leading to the release of significant amounts of Mg^2+^, which is known to inhibit this enzyme [106].

The observed decrease in LDH activity, a key enzyme in the anaerobic metabolic pathway [103], is also likely related to the destruction of red blood cells and the development of hypoxia. In particular, a decrease in LDH activity was demonstrated in Amur goby (*Rhynchocypris lagowskii*) and *Rhinelepis strigosa* under induced hypoxia [107,108]. This study also revealed a significant decrease in the red blood cell count and abundant cell debris in blood smears, indicating, indicating hemolysis and concomitant hypoxia. Typically, an increase in the activity of these enzymes is associated with extensive damage to internal organs, in particular, muscle tissue [109]. For example, Mbokane et al. [98] described an increase in ALP and LDH activity in Mozambican tilapia (*Oreochromis mossambicus*) at 1 DPI after infection with *A. hydrophila*. Thus, the decrease in enzyme activity observed in this study may represent a specific stress response to the systemic spread of an infectious agent with pronounced hemolytic activity.

Histopathological changes in the examined organs were mainly characterized by the development of congestive phenomena and microcirculation disorders, including capillary dilation and vascular stasis. Degenerative changes detected in the liver (foci of hepatocyte necrosis) and kidneys (glomerular hypercellularity, foci of inflammation of the hematopoietic tissue) indicate tissue damage caused by the cytotoxic action of bacterial toxins and other extracellular products, including hemolysin, aerolysin, and enterotoxin, which are characteristic products of *A. salmonicida* Sl0n and other representatives of the genus *Aeromonas* [110,111,112]. Primary disorders in muscle tissue (myomer degradation) are probably more related to the mechanical effect of the injection, as they were also observed in control individuals. Similar histopathological changes in various organs have been described in studies of the pathogenesis of bacterial diseases caused by pathogenic *Aeromonas spp*. between 1 and 3 DPI [14,62,84,102,113].

The most significant changes during this period were observed in the spleen tissues, confirming its role as the primary target organ [14,114]. The increase in the size of reticuloendothelial cells is likely associated with the accumulation of a significant amount of antigens in the lumen of the sinusoids. In particular, Coscelli et al. [33] used immunohistochemical methods to demonstrate that infection of turbot (*Scophthalmus maximus*) with *A. salmonicida* causes antigen accumulation near the ellipsoids. Ellipsoids, being the terminal branches of the splenic arterioles, filter blood plasma, retaining immune complexes [115]. The invasion of the pathogen into the bloodstream led to an increase in the filtration activity of ellipsoid macrophages, leading to their increased size. PAS staining showed that foreign bodies localized in the cytoplasm of macrophages and the reticular network stained positive, which was previously noted by Espenes et al. [116]. Similar structural abnormalities of ellipsoids were found in channel catfish (*Ictalurus punctatus*) infected with *A. hydrophila* [113].

### 4.4. Second Stage of Pathogenesis (4 DPI)

The most pronounced clinical manifestations and pathological changes in the examined organs were observed at 4 DPI. The clinical signs of the disease at this stage of pathogenesis were similar to those in the acute phase, demonstrating a typical picture of septicemia [14,116,117], characterized by marked inflammation of the internal organs and swelling at the injection site. However, the bacterial load in the organs was significantly lower than at 2 DPI. Such dynamics are characteristic of moderately virulent or avirulent strains that are incapable of long-term host persistence [14,36].

Hematological analysis on day 4 revealed a robust immune response. The leukocyte count at 4 DPI was characterized by a predominance of neutrophils and a significant decrease in the number of monocytes and basophils compared to 2 DPI, although their levels remained above control values. Neutrophils play a key role in the immune response to various infectious agents due to their ability to phagocytose cells and foreign particles, as well as produce superoxide anions and bactericidal substances [118]. Neutrophilia combined with leukocytosis is a typical response of the host to the systemic spread of a pathogen and is a well-documented response in the literature [101,119]. The observed decrease in the relative number of monocytes and basophils at 4 DPI may indicate a transition from the acute phase of the inflammatory response to pathogen invasion to a more stable stage, typified by inflammation within target organs [120]. This is consistent with the histological examination, which revealed a marked inflammatory reaction in muscle and posterior intestine tissue, characterized by significant leukocyte infiltration.

A significant decrease in red blood cell count was also observed, accompanied by an increase in immature cell forms. One of the key factors of *A. salmonicida* virulence is its ability to produce hemolysins and aerolysins, which induce the destruction of host red blood cells [37,121], leading to a decrease in the oxygen-carrying capacity of the blood and the development of anemia. The hemolytic activity of the studied strain is confirmed by its cultivation on blood agar, the deposition of hemosiderin in hematopoietic organs and the liver, and the detection of significant cell debris characteristic of hemolyzed erythrocytes in peripheral blood smears. Furthermore, the detection of the *ahh1*, *hlyA*, and *ast* genes encoding hemolysins in the studied strain directly corroborates these findings. The previously observed (1–2 DPI) decrease in ALP activity persisted at 4 DPI, further indicating erythrocyte hemolysis. It is noteworthy that LDH activity, conversely, increased substantially compared to the earlier period and exceeded control values. This may be explained by the enzyme’s release due to organ damage, particularly in the liver and muscles [98,122].

The blood’s biochemical profile showed significant changes across most of the parameters studied. Specifically, at 4 DPI, a marked decrease in globulin (GLO) levels was detected compared to both the control group and the 2 DPI infected group. Previous studies have shown that a decrease in total protein and its fractions is a characteristic response to infection with various infectious agents. Such changes were observed in a sturgeon hybrid (*Acipenser baerii* ♀ × *Acipenser schrencki* ♂) infected with *Citrobacter freundii* [122], Atlantic salmon (*Salmo salar*) infected with *A. salmonicida* [44], and black sea bass (*Centropristis striata*) infected with *Vibrio harveyi* [123]. A decrease in blood protein levels is traditionally associated with blood loss and impaired protein synthesis in the liver [103,124]. A decrease in blood oncotic pressure, a common consequence of severe infection, leads to increased vascular permeability and exudate formation. This process likely contributed to the fluid accumulation observed in the abdominal cavity of the infected fish. This assumption is further confirmed by the identified disorders and morphological changes within kidney tissue, indicating decreased functional activity and protein reabsorption capacity at 4 DPI.

A significant increase in the activity of aminotransferases (AST and ALT), which are biomarkers of liver damage [125], correlated with the detection of extensive foci of hepatocyte necrosis in infected fish. It is noteworthy that these foci of necrosis did not show infiltration by immune cells but predominantly exhibited morphological signs of apoptosis, indicating direct cytotoxic damage to hepatocytes. This type of cell death may be due to the presence of a type II secretion system (T2SS) in the strain and/or the action of specific toxins. It is generally accepted that the type III secretion system (T3SS), which was absent in the studied strain, is a key factor in virulence, as it ensures the direct translocation of bacterial toxins into the cytoplasm of the host cell [3]. In the case of the Sl0n strain, T2SS is responsible for the bulk secretion of toxins into the environment without attacking individual cells [126]. However, the complete absence of T3SS in the isolate suggests that under the conditions of acute septicemia observed in this study, the action of T2SS in combination with protective and immune evasion factors (ECA, RTX toxins) led to the development of widespread areas of liver tissue necrosis without pronounced signs of inflammation, which indicates a cytotoxic rather than inflammatory mechanism of cell death [127]. The presence of signs of hepatocyte apoptosis may also be associated with the activation of apoptosis signaling pathways (e.g., NF-κB and Caspase 3) and the blocking of pro-inflammatory signals, resulting in apoptosis without an accompanying inflammatory response. Studies have shown, that *A. hydrophila* induces the expression of Caspase 3 in rohu (*Labeo rohita*) [128], and *A. salmonicida* activates the NF-κB pathway during infection of golden carp [61] and lumpfish (*Cyclopterus lumpus*) [129]. Such extensive liver damage leads to disruption of metabolic, detoxification, and excretory functions of the body [130].

Increased LDH activity and creatinine concentration likely correlate with extensive damage to internal organs, confirmed both macroscopically and microscopically. Creatinine is the end product of muscle tissue metabolism, excreted mainly by the kidneys and gills [131]. Consequently, an elevated creatinine concentration is typically associated with renal dysfunction [103] and muscle tissue injury [109], which aligns with the findings of this study. The most pronounced degenerative changes in muscle tissue were observed on day 4 post-infection, which is consistent with the results of Sharifpour et al. [45].

The changes identified in the mesonephros tissue (edema, glomerular hypercellularity, parietal epithelial hypertrophy) indicate partial organ dysfunction. Notably, morphometric data revealed an increase in the area of glomerular capillaries, renal corpuscles, and distal tubule epithelium, which can be interpreted as a morphological manifestation of compensatory organ adaptation in response to the loss of some functional nephrons [42]. The pathological changes in the kidneys were notably less pronounced than in other organs, an atypical finding for bacterial infections. The kidney is widely recognized as one of the key target organs for this pathogen [132]. In contrast, infections caused by other bacteria, such as *A. hydrophila* [113], *Pseudomonas truttae* [133], *Vibrio anguillarum* [134], and *A. salmonicida* [61], have resulted in more pronounced pathological changes, including extensive necrosis of the tubular epithelium and hematopoietic tissue, hemorrhages, vacuolization, and inflammation. The moderate pathogenic effect of the studied strain on kidney tissue may be associated with a unique set of virulence factors and the characteristics of their expression. This further confirms the diversity of the pathomorphological picture of furunculosis.

The pathological changes observed in the posterior intestine were consistent with acute enteritis, characterized by necrosis of the mucosa, extensive hyperemia, and inflammation of the submucosa. Extensive intestinal lesions indicate the ability of the SL0n strain to overcome the intestinal barrier, even though the most likely route of spread to the intestine and other organs is hematogenous. *A. salmonicida* can penetrate the intestinal barrier both transcellularly, through surface-bound proteins and secreted toxins [135], and paracellularly, due to disruption of intercellular contacts [136,137]. Consequently, the identified intestinal lesions are likely caused by a two-stage process: an initial disruption of the epithelial barrier, followed by the pathogenic effects of microbial colonization and virulence factor production, which ultimately results in toxemia. Additionally, it is plausible that the thermostable enterotoxin encoded by the *ast* gene, as well as aerolysin, play a key role in intestinal tissue damage [138]. Similar intestinal tissue disorders have been previously described in a number of studies [62,113,136,139].

It should also be noted that different intestinal sections react differently to infection with *Aeromonas* spp. For example, Ringø et al. [136] identified the anterior intestine of Atlantic salmon as the most likely route of infection and the site of the most significant pathological changes. In contrast, the posterior intestine exhibited a more pronounced response to the introduction of inactivated bacteria [135] and potentially faster recovery after infection compared to the anterior and middle sections [140]. In this study, the posterior intestine exhibited more pronounced macroscopic and microscopic damage, suggesting a greater susceptibility to the pathogenic effects of the investigated strain.

### 4.5. Third Stage of Pathogenesis (6 DPI)

By 6 DPI, the final observation point, the injected fish showed partial regression of pathological changes, though residual morphological and physiological abnormalities persisted. In particular, the leukocyte formula remained similar to that at 4 DPI, characterized by neutrophilia. The neutrophil count peaked at 6 DPI, with segmented forms predominating. A further increase in the number of neutrophils, coupled with a slight decrease in the total number of leukocytes, may indicate an increase in myelopoiesis to limit the spread and localization of infection [95]. This assumption is consistent with clinical and histopathological observations showing a decrease in the severity of inflammatory reactions in organs despite persistent tissue damage. This observed phenomenon—a decrease in inflammatory severity despite persistent tissue damage—aligns with stress responses in fish exposed to various toxic substances, indicating a potential immunosuppressive effect of the pollutant [141]. It is important to note that at 6 DPI, the bacterial load was detected exclusively in the liver and was significantly reduced compared to previous time points.

A significant increase in immature red blood cells may be associated with a decrease in the pathogen’s hemolytic activity. This assumption is confirmed by a marked increase in ALP activity and the restoration of total bilirubin concentration to control values. It has previously been shown that erythropoiesis activation, evidenced by an increase in immature erythrocytes in peripheral blood, is a typical compensatory response to erythrocyte damage [70,142]. A similar restoration of erythrocyte parameters during late stages of infectious diseases was observed in sturgeon hybrids infected with *C. freundii* [122] and in Nile tilapia infected with *A. hydrophila* [102].

Despite signs of partial recovery in some hematological and biochemical parameters, the activity of aminotransferase enzymes (AST and ALT), as well as creatinine and glucose concentrations, remained significantly elevated in infected individuals compared to controls. These results are consistent with histological findings, which revealed extensive foci of hepatocyte necrosis and the presence of pyknotic apoptotic bodies in the spleen, along with marked organ hyperemia. This demonstrates that while the host was beginning to control the infection, the resolution of severe organ damage was far from complete by 6 DPI. Previous studies have shown that liver dysfunction in infectious diseases persists the longest [101,143]. This is likely due to the key role these organs play in detoxifying the body and maintaining the immune response; consequently, damage resolution occurs much more slowly due to pronounced functional and structural stress.

By 6 DPI, regression of the severity and spread of pathological changes was evident in muscle tissue, along with signs of restoration of tissue structural integrity, characterized by the growth of connective tissue accompanied by leukocyte infiltration. These data confirm the pathogen’s localization and are consistent with the results of Sharifpour et al. [45], who showed the development of fibrosis in the muscle tissue of the carp 4–6 days after intramuscular infection with *A. hydrophila*, and with those of Roy et al. [144], who recorded the onset of wound healing in Nile tilapia 6–8 days after infection with *A. caviae*. This pathogen localization and the initiation of healing processes at the studied dose of strain SL0n confirm its moderate virulence.

Partial regression of pathological changes was also noted in the renal parenchymal tissue, particularly a reduction in the severity of edema and a decrease in the size of the glomeruli and Bowman’s space. Simultaneously, a statistically significant decrease in the height of the proximal tubule epithelium was observed, along with signs of hyaline degeneration. Hyaline degeneration of the proximal renal tubules is a characteristic sign of increased proteinuria, where intracellular accumulation of proteinaceous material occurs in the epithelium [145]. Similar disorders have previously been described in bacterial infections in channel catfish [113] and rainbow trout [133]. The main cause of this pathology is damage to Bowman’s capsule, evident in the organ from 2 to 6 DPI, which can lead to the penetration of plasma proteins (albumin and globulins) into the primary filtrate and their subsequent increased reabsorption in the proximal tubules [42]. Notably, only a statistically significant decrease in globulin levels was detected in the blood of infected fish, while the concentration of total protein and albumin remained at control levels. This may explain why the development of hyaline degeneration was recorded only at 6 DPI.

The condition of the posterior intestine at 6 DPI reflected the completion of the active phase of bacterial invasion. Despite the presence of areas of desquamation and epithelial necrosis, signs of acute inflammation, previously observed at 4 DPI, were no longer evident. Simultaneously, the preserved areas of the mucous membrane exhibited fewer goblet cells and intraepithelial leukocytes, along with signs of mucosal edema likely stemming from an acute inflammatory reaction affecting all intestinal tissues. During the development of the immune response, inflammatory mediators led to an increase in the permeability of the capillaries of the submucosa and muscularis, causing interstitial edema [146]. This edema persisted until 6 DPI, as the restoration of vascular permeability and normalization of plasma drainage are more protracted processes. Structural disruption and edema of the muscle layer during infection with pathogenic bacteria were previously noted by Liu et al. [122] and Abdelhamed et al. [113]. These results indicate that despite clinical improvement in intestinal tissues, complete morphofunctional restoration of the intestinal barrier requires significantly more time.

A decrease in the overall prevalence of hemodynamic disorders and a reduction in the hypotrophy of reticular endothelial cells, coupled with the appearance of connective tissue nodules in the spleen, indicates a degree of organ recovery. The presence of numerous apoptotic cells may also indicate the infection resolution stage, in which programmed cell death within the spleen affects both pathogen-damaged cells and immune cells involved in the response or undergoing normal turnover after activation [147]. This cellular debris is cleared by phagocytic cells of the spleen [148]. Coupled with a reduction in other pathological changes, this suggests a restoration of the infected fish’s clearance system function by 6 DPI.

### 4.6. Dynamics of Physiological and Pathological Disorders Induced by A. salmonicida SL0n

Analysis of the pathological dynamics in rainbow trout infected with a sublethal dose of the moderately virulent *A. salmonicida* strain SL0n revealed an atypical clinical picture compared to that of acute, high-dose experiments or the previously described chronic form of the disease, yet it was still consistent with systemic infection. Nevertheless, the results indicate the development of a systemic septic process in the fish. Thus, despite the absence of typical clinical signs, the observed pathogenesis shares characteristics with the acute form of furunculosis.

A comprehensive analysis of the data reveals three consecutive stages in the pathogenesis of the disease induced by the studied *A. salmonicida* SL0n: (i) an early stage (1–2 DPI), or stress response, representing the body’s initial reaction to infection via the activation of adaptive defense mechanisms, evidenced by changes in hematological parameters and blood biochemistry; (ii) an acute stage (3–4 DPI), or acute bacteremia, characterized by the most severe pathomorphological damage and organ dysfunction; and (iii) a recovery stage (from 6 DPI onwards), during which pathological changes subside and regenerative processes commence. Notably, the described stages of infection development are not universal but represent a conceptual framework for interpreting the data from this specific study; their duration and the severity of pathological abnormalities may vary depending on the microorganism’s strain, as well as the host organism’s species and physiological state.

The early stage of the disease is characterized by the spread of bacteria in the fish’s body, as they overcome the host’s barriers due to the presence of specific virulence factors. In particular, adhesion systems play a key role in this process. The presence of polar flagella, type IV pili (*Flp* and *Tap*), mannose-sensitive hemagglutinins (*Msh*), and type I fimbriae in the SL0n strain ensures effective attachment of bacteria to muscle cells and vascular endothelium, contributing to their further spread [149]. The significant spread of bacteria in the fish’s body (peak at 2 DPI) was associated with both the development of an immune response (mobilization of phagocytic elements of the blood) and primary organ damage (myocyte degradation, edema, dilatation of sinusoidal capillaries in the liver, and splenic hyperemia This may be due to T2SS, the main export mechanism for exotoxins in the studied strain [93].

In the acute stage of infection, despite a decrease in the bacterial load, accumulated toxins reach critical concentrations that lead to the most severe pathological changes in organs. In particular, RTX toxins and aerolysin cause extensive necrosis and apoptosis in target organs [150], while hemolysins (*ahh1*, *hlyA*, *hly-III*, *TH*) lead to massive destruction of red blood cells [84]. Acute enteritis is probably caused by the secretion of enterotoxin (*Act*) by the strain, which, in combination with other toxins, causes severe toxemia [151]. An important feature is the absence of T3SS in this strain; consequently, toxins are secreted in large quantities into the environment (via T2SS) rather than injected into individual cells. This likely explains the development of extensive, diffuse necrotic changes in the liver, often without pronounced leukocyte infiltration, which may indicate a cytotoxic rather than inflammatory mechanism of cell death [127].

The recovery stage is characterized by the immune system regaining control over the infection, evidenced by a significantly reduced bacterial load and high levels of segmented neutrophils that clear the remaining bacteria and their decay products. It is likely that the presence of a capsule similar to Acinetobacter (*fdtA–fdtC*, *rmlA*) in the strain allows the pathogen to effectively evade the host’s immune system only at the initial stage of infection [151]. The absence in SL0n of a complete *wecA–G* operon encoding the common antigen of enterobacteria [152], as well as other genes involved in immune evasion (A-layer, serine protease, lipopolysaccharides, T3SS), characteristic of highly virulent strains of *A. salmonicida* [25], allows the organism to partially halt the spread of infection and initiate regenerative processes (increase in the number of immature erythrocytes, fibrosis of muscle tissue, reduction in intestinal inflammation). At the same time, the wide range of toxins produced by the strain and released via T2SS resulted in significant organ and tissue damage (liver necrosis, glomerular hypercellularity and hyaline degeneration), from which recovery may be prolonged. The recovery phase after infection—namely, the repair and restoration of internal organ functions in fish—is the least characterized in the literature.

Thus, the molecular basis for the observed pathogenesis can be directly linked to the genomic profile of strain SL0n. The profound anemia and hemolysis are explained by a genomic arsenal encoding multiple hemolysins (*ahh1*, *hlyA*, *hly-III*, *TH*). The severe cytotoxic damage, observed as diffuse necrosis in the liver and acute enteritis in the gut, is consistent with the action of potent toxins like aerolysin (*aerA*) and enterotoxin (*Act*), which are secreted into the extracellular space by the strain’s T2SS. The extensive pathogen distribution in the organism, observed by the increase in bacterial load at 2 and 4 DPI, is associated with the presence of adhesion systems (type IV pili, fimbriae).

Despite the significant amount of material studied, this research has several limitations. The study of the pathogenesis of infectious diseases in fish and other aquatic organisms is associated with numerous methodological difficulties that prevent a complete characterization of the disease stages. The key problem lies in the significant individual variability of the response to infection in aquatic organisms, which precludes random sampling of individuals for analysis. In this study, we used targeted selection of individuals with the most pronounced clinical signs, which intentionally introduced a selection bias to characterize the full extent of pathogenesis but limits the generalizability of the observed severity to the entire infected population. An alternative approach involves analyzing all individuals regardless of clinical signs, followed by determining the disease stage based on histological and physiological data. For example, this approach was used in a study by Dils et al. [153] on the dynamics of histopathological changes in Chinook salmon (*Oncorhynchus tschawytscha*) infected with *Renibacterium salmoninarum*. Targeted selection of muscle tissue, liver, posterior intestine, kidneys, and spleen for in-depth study allowed for a detailed characterization of the key damage mechanisms in these organs caused by A. salmonicida SL0n. Subsequent studies will focus on expanding the list of organs examined to verify the systemic nature of the identified disorders.

In addition, the morphophysiological features of disease pathogenesis described in this study serve as an essential basis for further in-depth research focused on assessing the complex molecular biological changes induced by *A. salmonicida*. A thorough assessment of the disease’s phenotypic manifestations, combined with an analysis of cellular, molecular, and genetic changes, contributes to a more comprehensive understanding of the mechanisms of disease development, as demonstrated in several studies [23,61].

## 5. Conclusions

This study comprehensively characterized the pathogenesis of acute infection in rainbow trout caused by a moderately virulent strain of *Aeromonas salmonicida* SL0n (LD50 = 1.63 × 10^6^ CFU/individual). The results allowed us to identify three consecutive stages of the disease:

- Early stage (1–2 DPI): Systemic spread of bacteria in the fish’s body and activation of a nonspecific immune response (leukocytosis, neutrophilia).
- Acute stage (4 DPI): Peak septicemia accompanied by severe clinical signs, anemia, and maximum organ damage (indicated by peak AST, ALT, and creatinine levels).
- Recovery stage (6 DPI): Partial regression of pathological changes and activation of regenerative processes, while maintaining pronounced organ dysfunction.

Whole-genome analysis of the SL0n strain confirmed its identity as *A. salmonicida* and revealed the presence of various virulence factors, including adhesion genes, toxins, and the type II secretion system (T2SS). Notably, the absence of plasmids and the type III secretion system (T3SS) likely determines the specificity of its pathogenesis, potentially warranting its classification as a distinct subspecies. This genomic profile correlates with the observed pathological picture, explaining the development of systemic infection and the nature of tissue damage resulting from exotoxin action.

## Figures and Tables

**Table 1:** Bacterial load of *A. salmonicida* in the internal organs of injected rainbow trout in a prolonged experiment.

Parameter	0 DPI	1 DPI	2 DPI	4 DPI	6 DPI
Blood, lg(CFU/mL)	0	1.36 ± 0.07	3.73 ± 0.05	1.49 ± 0.04	0
Liver, lg(CFU/g)	0	3.09 ± 0.04	5.51 ± 0.05	2.66 ± 0.04	1.68 ± 0.11
Muscule, lg(CFU/g)	0	0	4.95 ± 0.07	2.33 ± 0.04	0

## Data Availability

The raw data supporting the conclusions of this article will be made available by the authors on request.

## Supplementary Materials

The following supporting information can be downloaded at: https://www.mdpi.com/article/10.3390/biology14101330/s1. Table S1: Measured histomorphometric parameters; Table S2: Biochemical, physiological characteristics and antibiotic sensitivity of SL0n isolate; Table S3: Prophages of A. salmonicida SL0n strain and their characteristics; Figure S1: Survival curve of rainbow trout at 96 HPI (Hours Post Injection) and (b) Kaplan–Meier survival curve when infected with different doses of A. salmonicida SL0n in an acute experiment (n = 20); Figure S2: Appearance of rainbow trout at 96 HPI with various doses of A. salmonicida SL0n in an acute experiment. Scale bar 2 cm; Figure S3: Condition of the trunk kidney in injected rainbow trout at 4 days post-injection (4 DPI); Figure S4: Condition of skeletal muscle (transverse plane) in injected rainbow trout at various experimental days; Table S4 Virulence gene profile of Aeromonas salmonicida SL0n strain compared to other Aeromonas strains; Table S5: Resistance gene profile of Aeromonas salmonicida SL0n strain; Table S6: Hematological parameters of rainbow trout in control and infected groups throughout a chronic experiment.; Table S7: Exact p-values for the studied parameters within a group across different time points and between the control (CTR) and experimental (AS) groups at a given time point.; Table S8: Serum biochemical parameters of rainbow trout in control and infected group at different time points; Figure S5: Histologic sections of dorsal musculature of control rainbow trout individuals at 1 (a) and 4 DPI (b). Abbreviations: MS—myosepta, MD—myomer degeneration, He—hemorrhage, RF—reticular fiber. H&E staining, scale bar 100 (a), 25 (b).; Table S9: Histomorphometric parameters of tissue elements of the posterior intestine of rainbow trout in the control and infected groups; Table S10: Histomorphometric parameters of tissue elements of the liver of rainbow trout in the control and infected groups.; Table S11: Histomorphometric parameters of tissue elements of the trunk kidney of rainbow trout in the control and infected groups; Table S12: Abbreviation of histopathological indexes and morphometric parameters used in the work.

## References

[1] Olivier G., Bernoth E.-M., Ellis A.E., Midtlyng P.J., Olivier G., Smith P. (1997). Getting to know your enemy. Furunculosis: Multidisciplinary Fish Disease Research.

[2] Austin B., Austin D.A. (2016). Aeromonadaceae representatives (*Aeromonas salmonicida*). Bacterial Fish Pathogens: Disease of Farmed and Wild Fish.

[3] Dallaire-Dufresne S., Tanaka K.H., Trudel M.V., Lafaille A., Charette S.J. (2014). Virulence, genomic features, and plasticity of *Aeromonas salmonicida* subsp. *salmonicida*, the causative agent of fish furunculosis. Vet. Microbiol..

[4] Parte A.C., Sardà Carbasse J., Meier-Kolthoff J.P., Reimer L.C., Göker M. (2020). List of prokaryotic names with standing in nomenclature (LPSN) moves to the DSMZ. Int. J. Syst. Evol. Microbiol..

[5] Alghabshi A., Austin B., Crumlish M. (2018). *Aeromonas salmonicida* isolated from wild and farmed fish and invertebrates in Oman. Int. Aquat. Res..

[6] Thomas J., Jerobin J., Seelan T.S.J., Thanigaivel S., Vijayakumar S., Mukherjee A., Chandrasekaran N. (2013). Studies on pathogenicity of *Aeromonas salmonicida* in catfish *Clarias batrachus* and control measures by neem nanoemulsion. Aquaculture.

[7] Treasurer J.W., Birkbeck T.H., Laidler L.A., Cox D.I. (2007). Atypical *Aeromonas salmonicida* infection in naturally- and laboratory-challenged farmed haddock, *Melanogrammus aeglefinus* (L.). J. Fish Dis..

[8] Bakiyev S., Smekenov I., Zharkova I., Kobegenova S., Sergaliyev N., Absatirov G., Bissenbaev A. (2023). Characterization of atypical pathogenic *Aeromonas salmonicida* isolated from a diseased Siberian sturgeon (*Acipenser baerii*). Heliyon.

[9] Pereiro P., Falcó A., Fernández-Oliver M., Paladea-Rojo R., Bonet-García R., Yuste J.E., Novoa B. (2025). Identification of taurine as a resistance-associated metabolite against *Aeromonas salmonicida* and its protective, immune-regulatory, and microbiota-shaping effects in turbot (*Scophthalmus maximus*). Aquaculture.

[10] Menanteau-Ledouble S., Kumar G., Saleh M., El-Matbouli M. (2016). *Aeromonas salmonicida*: Updates on an old acquaintance. Dis. Aquat. Org..

[11] Guo Y., Zheng C., Wang Y., Dang Y., Li R., Tao Y., Yang Y., Sun X., Song Z., Sun P. (2025). Study on the role and pathological and immune responses of silver nanoparticles against two *Aeromonas salmonicida* subsp. *salmonicida* strains at different virulence levels in rainbow trout (*Oncorhynchus mykiss*). Fishes.

[12] Boulanger Y., Lallier R., Cousineau G. (1977). Isolation of enterotoxigenic *Aeromonas* from fish. Can. J. Microbiol..

[13] Buller N.B. (2004). Bacteria from Fish and Other Aquatic Animals: A Practical Identification Manual.

[14] Marinho-Neto F.A., Claudiano G.S., Yunis-Aguinaga J., Cueva-Quiroz V.A., Kobashigawa K.K., Cruz N.R.N., Moraes F.R., Moraes J.R.E. (2019). Morphological, microbiological and ultrastructural aspects of sepsis by *Aeromonas hydrophila* in *Piaractus mesopotamicus*. PLoS ONE.

[15] Abreu R.E.F.F., Magalhães T.C., Souza R.C., Teixeira A.B., Moreira E.L.T., Rosa P.S., Pinheiro R.O., Alves D.B.M., Byrd D.S., Lemos M.L. (2018). Environmental factors on virulence of *Aeromonas hydrophila*. Aquac. Int..

[16] Gjerde B., Evensen Ø., Bentsen H.B., Storset A. (2009). Genetic (co)variation of vaccine injuries and innate resistance to furunculosis (*Aeromonas salmonicida*) and infectious salmon anaemia (ISA) in Atlantic salmon (*Salmo salar*). Aquaculture.

[17] Lund V., Mikkelsen H., Schrøder M.B. (2008). Comparison of atypical furunculosis vaccines in spotted wolffish (*Anarhicas minor* O.) and Atlantic halibut (*Hippoglossus hippoglossus* L.). Vaccine.

[18] Diamanka A., Loch T.P., Cipriano R.C., Faisal M. (2014). Polyphasic characterization of *Aeromonas salmonicida* isolates recovered from salmonid and non-salmonid fish. J. Fish Dis..

[19] Hiney M. (1999). Field observation of clinical furunculosis in Atlantic salmon smolts vaccinated with an oil-based furunculosis vaccine. Bull. Eur. Assoc. Fish Pathol..

[20] Gudding R., Van Muiswinkel W.B. (2013). A history of fish vaccination: Science-based disease prevention in aquaculture. Fish Shellfish Immunol..

[21] Ahmed I., Ishtiyaq S., Sayed S.F. (2025). An overview on understanding the major bacterial fish diseases in freshwater salmonids. Front. Aquac..

[22] Huang Y., Li Z., Li M., Zhang X., Shi Q., Xu Z. (2025). Fish genomics and its application in disease-resistance breeding. Rev. Aquac..

[23] Pereiro P., Tur R., García M., Figueras A., Novoa B. (2024). Unravelling turbot (*Scophthalmus maximus*) resistance to *Aeromonas salmonicida*: Transcriptomic insights from two full-sibling families with divergent susceptibility. Front. Immunol..

[24] Majeed S., De Silva L.A.D.S., Kumarage P.M., Heo G.-J. (2023). Occurrence of potential virulence determinants in *Aeromonas* spp. isolated from different aquatic environments. J. Appl. Microbiol..

[25] Reith M.E., Singh R.K., Curtis B., Boyd J.M., Bouevitch A., Kimball J., Munholland J., Murphy C., Sarty D., Williams J. (2008). The genome of *Aeromonas salmonicida* subsp. *salmonicida* A449: Insights into the evolution of a fish pathogen. BMC Genom..

[26] Sreedharan K., Philip R., Singh I.S.B. (2013). Characterization and virulence potential of phenotypically diverse *Aeromonas veronii* isolates recovered from moribund freshwater ornamental fishes of Kerala, India. Antonie Van Leeuwenhoek.

[27] Abdella B., Shokrak N.M., Abozahra N.A., Elshamy Y.M., Kadira H.I., Mohamed R.A. (2024). Aquaculture and *Aeromonas hydrophila*: A complex interplay of environmental factors and virulence. Aquac. Int..

[28] Vanden Bergh P., Frey J. (2014). *Aeromonas salmonicida* subsp. *salmonicida* in the light of its type-three secretion system. Microb. Biotechnol..

[29] Wiklund T., Dalsgaard I. (1998). Occurrence and significance of atypical *Aeromonas salmonicida* in non-salmonid and salmonid fish species: A review. Dis. Aquat. Org..

[30] Bernoth E.-M., Bernoth E.-M., Ellis A.E., Midtlyng P.J., Olivier G., Smith P. (1997). Diagnosis of furunculosis: The tools. Furunculosis: Multidisciplinary Fish Disease Research.

[31] Ogut H., Reno P.W. (2005). Evaluation of an experimental *Aeromonas salmonicida* epidemic in chinook salmon, *Oncorhynchus tshawytscha* (Walbaum). J. Fish Dis..

[32] Cipriano R.C., Bullock G.L. (2001). Furunculosis and Other Diseases Caused by *Aeromonas salmonicida*.

[33] Coscelli G.A., Bermúdez R., Losada A.P., Faílde L.D., Santos Y., Quiroga M.I. (2014). Acute *Aeromonas salmonicida* infection in turbot (*Scophthalmus maximus* L.). Histopathological and immunohistochemical studies. Aquaculture.

[34] Noga E.J. (2010). Fish Disease: Diagnosis and Treatment.

[35] Magnadóttir B., Bambir S.H., Gudmundsdóttir B.K., Pilström L., Helgason S. (2002). Atypical *Aeromonas salmonicida* infection in naturally and experimentally infected cod, *Gadus morhua* L.. J. Fish Dis..

[36] Farto R., Milton D.L., Bermúdez M.B., Nieto T.P. (2011). Colonization of turbot tissues by virulent and avirulent *Aeromonas salmonicida* subsp. *salmonicida* strains during infection. Dis. Aquat. Org..

[37] Han H.J., Kim D.Y., Kim W.S., Kim C.S., Jung S.J., Oh M.J., Kim D.H. (2011). Atypical *Aeromonas salmonicida* infection in the black rockfish, *Sebastes schlegeli* Hilgendorf, in Korea. J. Fish Dis..

[38] Basak C., Chakraborty R. (2024). Protocol to establish a disease model in *Lepidocephalichthys guntea* using *Aeromonas hydrophila*. STAR Protoc..

[39] Plumb J.A., Hanson L.A. (2011). Health Maintenance and Principal Microbial Diseases of Cultured Fishes.

[40] Daly J.G., Kew A.K., Moore A.R., Olivier G. (1996). The cell surface of *Aeromonas salmonicida* determines in vitro survival in cultured brook trout (*Salvelinus fontinalis*) peritoneal macrophages. Microb. Pathog..

[41] Lian Z., Bai J., Hu X., Lü A., Sun J., Guo Y., Song Y. (2020). Detection and characterization of *Aeromonas salmonicida* subsp. salmonicida infection in crucian carp *Carassius auratus*. Vet. Res. Commun..

[42] Bhattacharyya T., Bardhan A. (2025). Histopathological changes in teleost kidney as indicators of aetiopathogenic involvement. J. Environ. Inform. Lett..

[43] Bernet D., Schmidt H., Meier W., Burkhardt-Holm P., Wahli T. (1999). Histopathology in fish: Proposal for a protocol to assess aquatic pollution. J. Fish Dis..

[44] Yi M., Du Y., Chi L., Sun G., Li X., Liu Y. (2016). The impact of *Aeromonas salmonicida* infection on behaviour and physiology of Atlantic salmon *Salmo salar* L.. Aquac. Res..

[45] Sharifpour I., Rahimi Afzal Z., Hemati A., Saeidi Z. (2024). Histology of the inflammatory response of carp (*Cyprinus carpio* L.) to *Aeromonas hydrophila* infection. Sustain. Aquac. Health Manage. J..

[46] Bergey D.H., Holt J.G., Krieg N.R., Sneath P.H.A., Staley J.T., Williams S.T. (1994). Bergey’s Manual of Determinative Bacteriology.

[47] Bauer A.W., Kirby W.M.M., Sherris J.C., Turck M. (1966). Antibiotic susceptibility testing by a standardized single disk method. Am. J. Clin. Pathol..

[48] Whitman K.A. (2004). Finfish and Shellfish Bacteriology Manual: Techniques and Procedures.

[49] Andrews S. FastQC: A Quality Control Tool for High Throughput Sequence Data. http://www.bioinformatics.babraham.ac.uk/projects/fastqc/.

[50] Chen S., Zhou Y., Chen Y., Gu J. (2018). fastp: An ultra-fast all-in-one FASTQ preprocessor. Bioinformatics.

[51] Camacho C., Coulouris G., Avagyan V., Ma N., Papadopoulos J., Bealer K., Madden T.L. (2009). BLAST+: Architecture and applications. BMC Bioinf..

[52] Wick R.R., Judd L.M., Gorrie C.L., Holt K.E. (2017). Unicycler: Resolving bacterial genome assemblies from short and long sequencing reads. PLoS Comput. Biol..

[53] Tatusova T., DiCuccio M., Badretdin A., Chetvernin V., Nawrocki E.P., Zaslavsky L., Ostell J. (2016). NCBI prokaryotic genome annotation pipeline. Nucleic Acids Res..

[54] Meier-Kolthoff J.P., Carbasse J.S., Peinado-Olarte R.L., Göker M. (2022). TYGS and LPSN: A database tandem for fast and reliable genome-based classification and nomenclature of prokaryotes. Nucleic Acids Res..

[55] Richter M., Rosselló-Móra R., Glöckner F.O., Peplies J. (2016). JSpeciesWS: A web server for prokaryotic species circumscription based on pairwise genome comparison. Bioinformatics.

[56] Carattoli A., Zankari E., García-Fernández A., Larsen M.V., Lund O., Villa L., Hasman H. (2014). In silico detection and typing of plasmids using PlasmidFinder and plasmid multilocus sequence typing. Antimicrob. Agents Chemother..

[57] Arndt D., Grant J.R., Marcu A., Sajed T., Pon A., Liang Y., Wishart D.S. (2016). PHASTER: A better, faster version of the PHAST phage search tool. Nucleic Acids Res..

[58] Ross K., Varani A.M., Snesrud E., Huang H., Alvarenga D.O., Zhang J., Chandler M. (2021). TnCentral: A prokaryotic transposable element database and web portal for transposon analysis. mBio.

[59] Zhou S., Liu B., Zheng D., Chen L., Yang J. (2025). *VFDB* 2025: An integrated resource for exploring anti-virulence compounds. Nucleic Acids Res..

[60] Alcock B.P., Huynh W., Chalil R., Smith K.W., Raphenya A.R., Wlodarski M.A., McArthur A.G. (2023). CARD 2023: Expanded curation, support for machine learning, and resistome prediction at the comprehensive antibiotic resistance database. Nucleic Acids Res..

[61] Ling X.-D., Dong W.-T., Zhang Y., Qian X., Zhang W.-D., He W.-H., Zhao X.-X., Liu J.-X. (2019). Comparative transcriptomics and histopathological analysis of crucian carp infection by atypical *Aeromonas salmonicida*. Fish Shellfish Immunol..

[62] Bhat R.A.H., Thakuria D., Dubey M.K., Tandel R.S., Sharma P., Khangembam V.C., Tripathi G., Dash P., Sarma D. (2021). Lethal dose and histopathological alterations induced by *Aeromonas salmonicida* in experimentally challenged common carp, *Cyprinus carpio*. Microb. Pathog..

[63] Blaxhall P.C., Daisley K.W. (1973). Routine haematological methods for use with fish blood. J. Fish Biol..

[64] Grant K.R. (2015). Fish hematology and associated disorders. Vet. Clin. N. Am. Exot. Anim. Pract..

[65] Fijan N. (2002). Morphogenesis of blood cell lineages in channel catfish. J. Fish Biol..

[66] Kondera E. (2011). Haematopoiesis in the head kidney of common carp (*Cyprinus carpio* L.): A morphological study. Fish Physiol. Biochem..

[67] Nabi N., Ahmed I., Wani G.B. (2022). Hematological and serum biochemical reference intervals of rainbow trout, *Oncorhynchus mykiss* cultured in Himalayan aquaculture: Morphology, morphometrics and quantification of peripheral blood cells. Saudi J. Biol. Sci..

[68] Suvarna S.K., Layton C., Bancroft J.D. (2018). Bancroft’s Theory and Practice of Histological Techniques.

[69] Schindelin J., Arganda-Carreras I., Frise E., Kaynig V., Longair M., Pietzsch T., Preibisch S., Rueden C., Saalfeld S., Schmid B. (2012). Fiji: An open-source platform for biological-image analysis. Nat. Methods.

[70] Smorodinskaya S., Kochetkov N., Gavrilin K., Nikiforov-Nikishin D., Reznikova D., Vatlin A., Danilenko V. (2023). The effects of acute bisphenol A toxicity on the hematological parameters, hematopoiesis, and kidney histology of zebrafish (*Danio rerio*). Animals.

[71] Nikiforov-Nikishin A.L., Nikiforov-Nikishin D.L., Kochetkov N.I. (2023). Status of isolated *Coregonus peled* peled populations from mountain lakes of Altai according to histological indices and elemental composition of eye lens. Inland Water Biol..

[72] Oropesa A.L., Jiménez B., Fallola C., Pula H.J., Cuesta J.M., Gómez L. (2013). Histological alterations on the structure of the excretory renal system in tench (*Tinca tinca*) after exposure to 17-alpha-ethynylestradiol. Bull. Environ. Contam. Toxicol..

[73] Escaffre A.M., Kaushik S., Mambrini M. (2007). Morphometric evaluation of changes in the digestive tract of rainbow trout (*Oncorhynchus mykiss*) due to fish meal replacement with soy protein concentrate. Aquaculture.

[74] Healy M.J.R., Finney D.J. (1979). Statistical Method in Biological Assay.

[75] RStudio Team (2020). RStudio: Integrated Development Environment for R.

[76] R Core Team (2021). R: A Language and Environment for Statistical Computing.

[77] Kirov S.M., Tassell B.C., Semmler A.B., O’Donovan L.A., Rabaan A.A., Shaw J.G. (2002). Lateral flagella and swarming motility in *Aeromonas* species. J. Bacteriol..

[78] Kirov S.M., Castrisios M., Shaw J.G. (2004). *Aeromonas* flagella (polar and lateral) are enterocyte adhesins that contribute to biofilm formation on surfaces. Infect. Immun..

[79] Stuber K., Burr S.E., Braun M., Wahli T., Frey J. (2003). Type III secretion genes in *Aeromonas salmonicida* subsp. *salmonicida* are located on a large thermolabile virulence plasmid. J. Clin. Microbiol..

[80] Vasquez I., Hossain A., Gnanagobal H., Valderrama K., Campbell B., Ness M., Santander J. (2022). Comparative genomics of typical and atypical *Aeromonas salmonicida* complete genomes revealed new insights into pathogenesis evolution. Microorganisms.

[81] Akram M., Hafeez-ur-Rehman M., Abbas F., Altaf I., Kanwal S., Mobeen N., Anjum S., Sughra F. (2025). Identification, isolation and pathogenicity of *Aeromonas salmonicida* and histopathology of infected *Oncorhynchus mykiss* in Punjab and northern areas of Pakistan. J. Fish..

[82] Kozińska A., Figueras M.J., Chacon M.R., Soler L. (2002). Phenotypic characteristics and pathogenicity of *Aeromonas* genomospecies isolated from common carp (*Cyprinus carpio* L.). J. Appl. Microbiol..

[83] Sudheesh P.S., Al-Ghabshi A., Al-Mazrooei N., Al-Habsi S. (2012). Comparative pathogenomics of bacteria causing infectious diseases in fish. Int. J. Evol. Biol..

[84] Bari S.M., Islam M.M., Amina A., Khatun M., Shahabuddin A.M. (2024). Molecular identification, histopathology and antibiotic susceptibility profiling of *Aeromonas veronii* isolated from *Oreochromis niloticus* in Bangladesh. Vet. Med. Sci..

[85] Pradhan S.K., Devi R., Khan M.I.R., Kamilya D., Choudhury T.G., Parhi J. (2023). Isolation of *Aeromonas salmonicida* subspecies *salmonicida* from aquaculture environment in India: Polyphasic identification, virulence characterization, and antibiotic susceptibility. Microb. Pathog..

[86] Janda J.M., Abbott S.L. (2010). The genus *Aeromonas*: Taxonomy, pathogenicity, and infection. Clin. Microbiol. Rev..

[87] Long M., Fan H., Gan Z., Jiang Z., Tang S., Xia H., Lu Y. (2023). Comparative genomic analysis provides insights into taxonomy and temperature adaption of *Aeromonas salmonicida*. J. Fish Dis..

[88] Charette S.J. (2023). *Aeromonas salmonicida*: Genomics, taxonomy, diversity, pathogenesis, treatments and beyond. Microorganisms.

[89] Piotrowska M., Popowska M. (2015). Insight into the mobilome of *Aeromonas* strains. Front. Microbiol..

[90] Pereira C., Duarte J., Costa P., Braz M., Almeida A. (2022). Bacteriophages in the control of *Aeromonas* sp. in aquaculture systems: An integrative view. Antibiotics.

[91] Izumikawa K., Ueki N. (1997). Atypical *Aeromonas salmonicida* infection in cultured Schlegel’s black rockfish. Fish Pathol..

[92] Mittal K.R., Lalonde G., Leblanc D., Olivier G., Lallier R. (1980). *Aeromonas hydrophila* in rainbow trout: Relation between virulence and surface characteristics. Can. J. Microbiol..

[93] Korotkov K.V., Sandkvist M. (2019). Architecture, function, and substrates of the type II secretion system. EcoSal Plus.

[94] Peatman E., Mohammed H., Kirby A., Shoemaker C.A., Yildirim-Aksoy M., Beck B.H. (2018). Mechanisms of pathogen virulence and host susceptibility in virulent *Aeromonas hydrophila* infections of channel catfish (*Ictalurus punctatus*). Aquaculture.

[95] Seibel H., Baßmann B., Rebl A. (2021). Blood will tell: What hematological analyses can reveal about fish welfare. Front. Vet. Sci..

[96] Rini R.K., Aisiah S., Nafisah L. (2024). Hematological and histological analysis of tilapia (*Oreochromis niloticus*) cultured in floating net cages after disease outbreak. J. Penelit. Pendidik. IPA.

[97] Korni F.M., EL-Nahass E.S., Ahmed W. (2017). An outbreak of motile *Aeromonas* septicemia in cultured Nile tilapia, *Oreochromis niloticus* with reference to hematological, biochemical and histopathological alterations. J. Fish Pathol..

[98] Mbokane E.M., Moyo N.A. (2018). Alterations of haemato-biochemical parameters pre and post-challenge with *Aeromonas hydrophila* and survival of *Oreochromis mossambicus* fed *Moringa oleifera*-based diets. Fish Shellfish Immunol..

[99] Bennett C.M., Kanki J.P., Rhodes J., Liu T.X., Paw B.H., Kieran M.W., Look A.T. (2001). Myelopoiesis in the zebrafish, *Danio rerio*. Blood.

[100] Carriero M.M., Mendes Maia A.A., Sousa R.L.M., Henrique-Silva F. (2016). Characterization of a new strain of *Aeromonas dhakensis* isolated from diseased pacu fish (*Piaractus mesopotamicus*) in Brazil. J. Fish Dis..

[101] Cueva-Quiroz V.A., Yunis-Aguinaga J., Ramos-Espinoza F.C., de Moraes F.R., de Moraes J.R.E. (2020). Acute hypercortisolemia inhibits innate immune parameters in *Piaractus mesopotamicus* experimentally infected with *Aeromonas hydrophila*. Aquaculture.

[102] Sharon J., Abraham T.J., Sen A., Das R., Sinha P., Boda S., Patil P.K. (2025). Haemato-biochemistry, erythromorphology, and histopathology of *Oreochromis niloticus* as influenced by *Aeromonas hydrophila* infection and florfenicol therapy. Anim. Res. One Health.

[103] Bojarski B., Witeska M., Kondera E. (2025). Blood biochemical biomarkers in fish toxicology—A review. Animals.

[104] Řehulka J. (1998). The blood indices of the rainbow trout, *Oncorhynchus mykiss* (Walbaum) in *Aeromonas*-induced ulcerous dermatitis. Acta Vet. Brno.

[105] Kulkarni R.S. (2017). Sex differences in the blood biochemical parameters of the fresh water fish, *Notopterus notopterus* (Pallas, 1789). World News Nat. Sci..

[106] Banaee M. (2020). Alkaline phosphatase activity as a biochemical biomarker in aqua-toxicological studies. Int. J. Aquat. Biol..

[107] Yang Y., Wang Z., Wang J., Lyu F., Xu K., Mu W. (2021). Histopathological, hematological, and biochemical changes in high-latitude fish *Phoxinus lagowskii* exposed to hypoxia. Fish Physiol. Biochem..

[108] Panepucci L., Fernandes M.N., Sanches J.R., Rantin F.T. (2000). Changes in lactate dehydrogenase and malate dehydrogenase activities during hypoxia and after temperature acclimation in the armored fish, *Rhinelepis strigosa* (*Siluriformes*, *Loricariidae*). Rev. Bras. Biol..

[109] Yousaf M.N., Powell M.D. (2012). The effects of heart and skeletal muscle inflammation and cardiomyopathy syndrome on creatine kinase and lactate dehydrogenase levels in Atlantic salmon (*Salmo salar* L.). Sci. World J..

[110] Chopra A.K., Xu X.J., Ribardo D., Gonzalez M., Kuhl K., Peterson J.W., Houston C.W. (2000). The cytotoxic enterotoxin of *Aeromonas hydrophila* induces proinflammatory cytokine production and activates arachidonic acid metabolism in macrophages. Infect. Immun..

[111] Beaz-Hidalgo R., Figueras M.J. (2013). *Aeromonas* spp. whole genomes and virulence factors implicated in fish disease. J. Fish Dis..

[112] Afifi S.H., Al-Thobiati S., Hazaa M.S. (2000). Bacteriological and histopathological studies on *Aeromonas hydrophila* infection of Nile tilapia (*Oreochromis niloticus*) from fish farms in Saudi Arabia. Assiut Vet. Med. J..

[113] Abdelhamed H., Ibrahim I., Baumgartner W., Lawrence M.L., Karsi A. (2017). Characterization of histopathological and ultrastructural changes in channel catfish experimentally infected with virulent *Aeromonas hydrophila*. Front. Microbiol..

[114] Bach R., Chen P.K., Chapman G.B. (1978). Changes in the spleen of the channel catfish *Ictalurus punctatus* Rafinesque induced by infection with *Aeromonas hydrophila*. J. Fish Dis..

[115] Sales C.F., Silva R.F., Amaral M.G., Domingos F.F., Ribeiro R.I., Thomé R.G., Santos H.B. (2017). Comparative histology in the liver and spleen of three species of freshwater teleost. Neotrop. Ichthyol..

[116] Espenes A., Press C.M.L., Dannevig B.H., Landsverk T. (1995). Investigation of the structural and functional features of splenic ellipsoids in rainbow trout (*Oncorhynchus mykiss*). Cell Tissue Res..

[117] Harikrishnan R., Balasundaram C. (2005). Modern trends in *Aeromonas hydrophila* disease management with fish. Rev. Fish. Sci..

[118] Mokhtar D.M., Zaccone G., Alesci A., Kuciel M., Hussein M.T., Sayed R.K.A. (2023). Main components of fish immunity: An overview of the fish immune system. Fishes.

[119] Zhang M., Xue M., Xiao Z., Liu W., Jiang N., Meng Y., Zhou Y. (2022). *Staphylococcus sciuri* causes disease and pathological changes in hybrid sturgeon *Acipenser baerii* × *Acipenser schrencki*. Front. Cell. Infect. Microbiol..

[120] Tort L. (2011). Stress and immune modulation in fish. Dev. Comp. Immunol..

[121] Wiklund T., Dalsgaard I., Eerola E., Olivier G. (1994). Characteristics of ‘atypical’, cytochrome oxidase-negative Aeromonas salmonicida isolated from ulcerated flounders (*Platichthys flesus* (L.)). J. Appl. Microbiol..

[122] Liu J., Ji K., Pang X., Jin S., Zheng Y., Xu J., Hu M. (2024). Effects of *Citrobacter freundii* on sturgeon: Insights from haematological and intestinal-liver immunity. Aquaculture.

[123] Mao X., Tian Y., Wen H., Liu Y., Sun Y., Yanglang A., Li Y. (2020). Effects of *Vibrio harveyi* infection on serum biochemical parameters and expression profiles of interleukin-17 (IL-17)/interleukin-17 receptor (IL-17R) genes in spotted sea bass. Dev. Comp. Immunol..

[124] Jumma S.Q., Shihab T.J., MahmoodHamad al-shammari S. (2022). Some hematological and biochemical parameter accompanying with *Aeromonas hydrophila* infection diagnosed genetically in *Cyprinus carpio*. Ann. Rom. Soc. Cell Biol..

[125] Shahjahan M., Islam M.J., Hossain M.T., Mishu M.A., Hasan J., Brown C. (2022). Blood biomarkers as diagnostic tools: An overview of climate-driven stress responses in fish. Sci. Total Environ..

[126] Fernández-Bravo A., Figueras M.J. (2020). An update on the genus *Aeromonas*: Taxonomy, epidemiology, and pathogenicity. Microorganisms.

[127] Aboyadak I.M., Soliman M.K., Nageeb H.M., Ali N.G. (2024). The role of *Aeromonas* genotyping in virulence for *Dicentrarchus labrax*. J. Fish Dis..

[128] Pal S., Roy D., Ray S.D., Homechaudhuri S. (2019). *Aeromonas hydrophila* induced mitochondrial dysfunction and apoptosis in liver and spleen of *Labeo rohita* mediated by calcium and reactive oxygen species. Turk. J. Fish. Aquat. Sci..

[129] Chakraborty S., Hossain A., Cao T., Gnanagobal H., Segovia C., Hill S., Santander J. (2022). Multi-organ transcriptome response of lumpfish (*Cyclopterus lumpus*) to *Aeromonas salmonicida* subspecies *salmonicida* systemic infection. Microorganisms.

[130] Khoshnood Z. (2017). Effects of environmental pollution on fish: A short review. Transylv. Rev. Syst. Ecol. Res..

[131] Baldissera M.D., Baldisserotto B. (2023). Creatine kinase activity as an indicator of energetic impairment and tissue damage in fish: A review. Fishes.

[132] Orozova P., Barker M., Austin D.A., Austin B. (2009). Identification and pathogenicity to rainbow trout, *Oncorhynchus mykiss* (Walbaum), of some aeromonads. J. Fish Dis..

[133] Oh W.T., Kim J.H., Jun J.W., Giri S.S., Yun S., Kim H.J., Park S.C. (2019). Genetic characterization and pathological analysis of a novel bacterial pathogen, *Pseudomonas tructae*, in rainbow trout (*Oncorhynchus mykiss*). Microorganisms.

[134] Ceylan M., Duman M., Inan S., Ozyigit O., Saticioglu I.B., Altun S. (2019). The study of histopathologic changes of experimental infection with *Listonella (Vibrio) anguillarum* in rainbow trout. J. Res. Vet. Med..

[135] Jutfelt F., Sundh H., Glette J., Mellander L., Björnsson B.T., Sundell K. (2008). The involvement of *Aeromonas salmonicida* virulence factors in bacterial translocation across the rainbow trout, *Oncorhynchus mykiss* (Walbaum), intestine. J. Fish Dis..

[136] Ringø E., Salinas I., Olsen R.E., Nyhaug A., Myklebust R., Mayhew T.M. (2007). Histological changes in intestine of Atlantic salmon (*Salmo salar* L.) following in vitro exposure to pathogenic and probiotic bacterial strains. Cell Tissue Res..

[137] Jutfelt F., Farrell A.P. (2011). Barrier function of the gut. Encyclopedia of Fish Physiology: From Genome to Environment.

[138] Sha J., Kozlova E.V., Chopra A.K. (2002). Role of various enterotoxins in *Aeromonas hydrophila*-induced gastroenteritis: Generation of enterotoxin gene-deficient mutants and evaluation of their enterotoxic activity. Infect. Immun..

[139] Ringø E., Jutfelt F., Kanapathippillai P., Bakken Y., Sundell K., Glette J., Olsen R.E. (2004). Damaging effect of the fish pathogen *Aeromonas salmonicida* ssp. *salmonicida* on intestinal enterocytes of Atlantic salmon (*Salmo salar* L.). Cell Tissue Res..

[140] Mu Q., Dong Z., Kong W., Wang X., Yu J., Ji W., Xu Z. (2022). Response of immunoglobulin M in gut mucosal immunity of common carp (*Cyprinus carpio*) infected with *Aeromonas hydrophila*. Front. Immunol..

[141] Witeska M., Kondera E., Bojarski B. (2023). Hematological and hematopoietic analysis in fish toxicology—A review. Animals.

[142] Kondera E., Bojarski B., Ługowska K., Kot B., Witeska M. (2020). Effects of oxytetracycline and gentamicin therapeutic doses on hematological, biochemical and hematopoietic parameters in *Cyprinus carpio* juveniles. Animals.

[143] Shaalan M., El-Mahdy M., Theiner S., Dinhopl N., El-Matbouli M., Saleh M. (2018). Silver nanoparticles: Their role as antibacterial agent against *Aeromonas salmonicida* subsp. salmonicida in rainbow trout (*Oncorhynchus mykiss*). Res. Vet. Sci..

[144] Roy A., Abraham T.J., Namdeo M.S., Singha J., Julinta R.B., Boda S. (2019). Effects of oral oxytetracycline-therapy on wound progression and healing following *Aeromonas caviae* infection in Nile tilapia (*Oreochromis niloticus* L.). Braz. Arch. Biol. Technol..

[145] Yancheva V., Velcheva I., Stoyanova S., Georgieva E. (2016). Histological biomarkers in fish as a tool in ecological risk assessment and monitoring programs: A review. Appl. Ecol. Environ. Res..

[146] Dale O.B., Tørud B., Kvellestad A., Koppang H.S., Koppang E.O. (2009). From chronic feed-induced intestinal inflammation to adenocarcinoma with metastases in salmonid fish. Cancer Res..

[147] Gogal R.M., Smith B.J., Kalnitsky J., Holladay S.D. (2000). Analysis of apoptosis of lymphoid cells in fish exposed to immunotoxic compounds. Cytometry.

[148] Zapata A.G. (2024). The fish spleen. Fish Shellfish Immunol..

[149] Masada C.L., LaPatra S.E., Morton A.W., Strom M.S. (2002). An *Aeromonas salmonicida* type IV pilin is required for virulence in rainbow trout *Oncorhynchus mykiss*. Dis. Aquat. Org..

[150] Gao S., Zhao N., Amer S., Qian M., Lv M., Zhao Y., Zhao B. (2013). Protective efficacy of PLGA microspheres loaded with divalent DNA vaccine encoding the ompA gene of *Aeromonas veronii* and the hly gene of *Aeromonas hydrophila* in mice. Vaccine.

[151] Tobin L.A., Jarocki V.M., Kenyon J., Drigo B., Donner E., Djordjevic S.P., Hamidian M. (2024). Genomic analysis of diverse environmental *Acinetobacter* isolates identifies plasmids, antibiotic resistance genes, and capsular polysaccharides shared with clinical strains. Appl. Environ. Microbiol..

[152] Maczuga N., Tran E.N., Qin J., Morona R. (2022). Interdependence of *Shigella flexneri* O antigen and enterobacterial common antigen biosynthetic pathways. J. Bacteriol..

[153] Dils R.E., Firestone T.B., Schaffer P.A., Winkelman D.L., Fetherman E.R. (2025). Histological progression and bacterial load dynamics of *Renibacterium salmoninarum* in Chinook salmon *Oncorhynchus tshawytscha*. Dis. Aquat. Org..

