# Supplementary material for: Dynamics of Pathomorphological and Pathophysiological Alterations in Rainbow Trout (Oncorhynchus mykiss) During Acute Aeromonas salmonicida Infection
Source: Biology (Basel). 2025 Sep 26;14(10):1330. doi: 10.3390/biology14101330 (PMC12561479; doi:10.3390/biology14101330)
Supplement: Supplementary file 1 [file biology-14-01330-s001.zip › Supplementary.pdf]

Table S1. Measured histomorphometric parameters

Organ	Structure	Parameter
**Liver**	Hepatocyte nuclei and cytoplasm	Area, perimeter, and diameter
	Lumen of sinusoidal capillaries	Width
	Hepatocytes	Density (cells/100 μm²)
**Posterior intestine**	Mucosal epithelium	Height
	Epithelial nuclei and goblet cells	Area
	Lamina propria, submucosa, muscularis, and serosa	Thickness
	Intraepithelial leukocytes and goblet cells	Number per 100 μm of mucosal length (cells/100 μm)
**Trunk kidney**	Glomerular capillaries, renal corpuscle, and capsular (Bowman's) space	Area
	Epithelium of the distal and proximal tubules	Thickness

Table S2. Biochemical, physiological characteristics and antibiotic sensitivity of SL0n isolate

Characteristics	Reaction	Antibiotics	Zone diameters, mm
Gram staining	N	Gentamicin	16
Morphology	Rod	Bacitracin	0
Motility	N	Ciprofloxacin	29
Oxidase	P	Ceftriaxone	7
Voges-Proskauer test	N	Chloramphenicol	12
OF-test	OF	Azithromycin	10
H2S formation	N	Cefixime	0
Indole formation	N	Tetracycline	4
Brown pigment	P		
Catalase	P		
Lactose	N		
Glycose acid	P		
Glycose gas	P		
13°C	P		
17°C	P		
27°C	P		
37°C	P		
0% NaCl	P		
1% NaCl	P		
2% NaCl	P		

Abbreviations: P - positive; N - negative; F – fermentative; O - oxidation.

Table S3. Prophages of A. salmonicida SL0n strain and their characteristics

Region	Contigs	Prophage characteristics			Possible Phage Match
		Region Position	Size, kb	Completeness	
1	SL0n_contig_5	95636-129860	34.2	Questionable	Salmonella phage ST64B (NC_004313)

2	SL0n_contig_9	103450-137672	34.2	Intact	Aeromonas phage phiO18P (NC_009542)

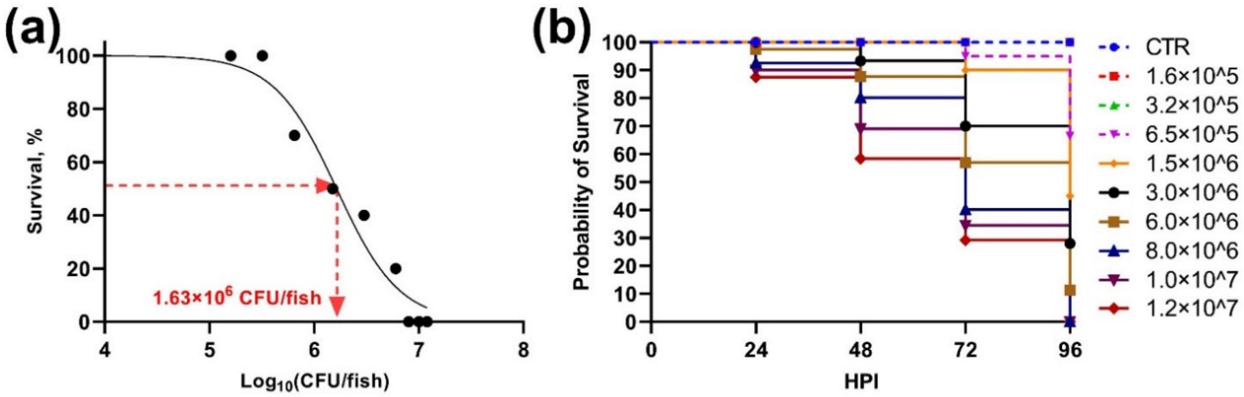

Figure S1. (a) Survival curve of rainbow trout at 96 HPI (Hours Post Injection) and (b) Kaplan-Meier survival curve when infected with different doses of *A. salmonicida* SL0n in an acute experiment (n = 20).

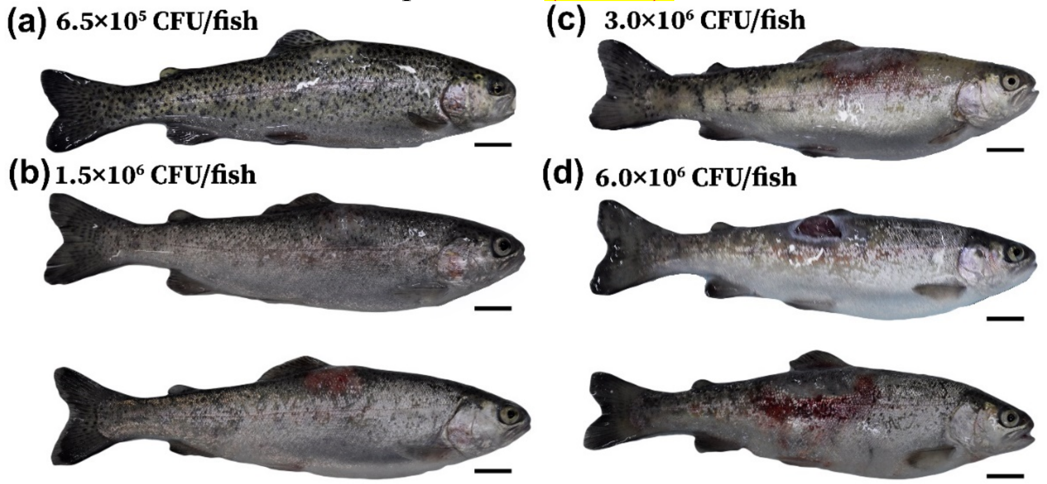

Figure S2. Appearance of rainbow trout at 96 HPI with various doses of *A. salmonicida* SL0n in an acute experiment. Scale bar 2 cm.

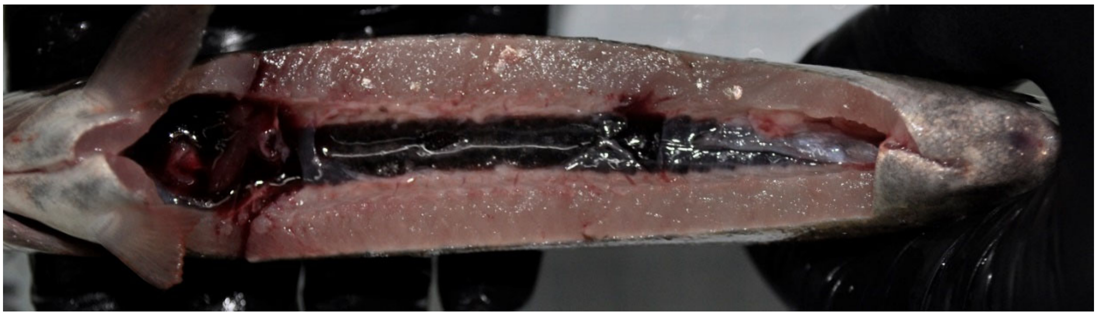

Figure S3. Condition of the trunk kidney in injected rainbow trout at 4 days post-injection (4 DPI).

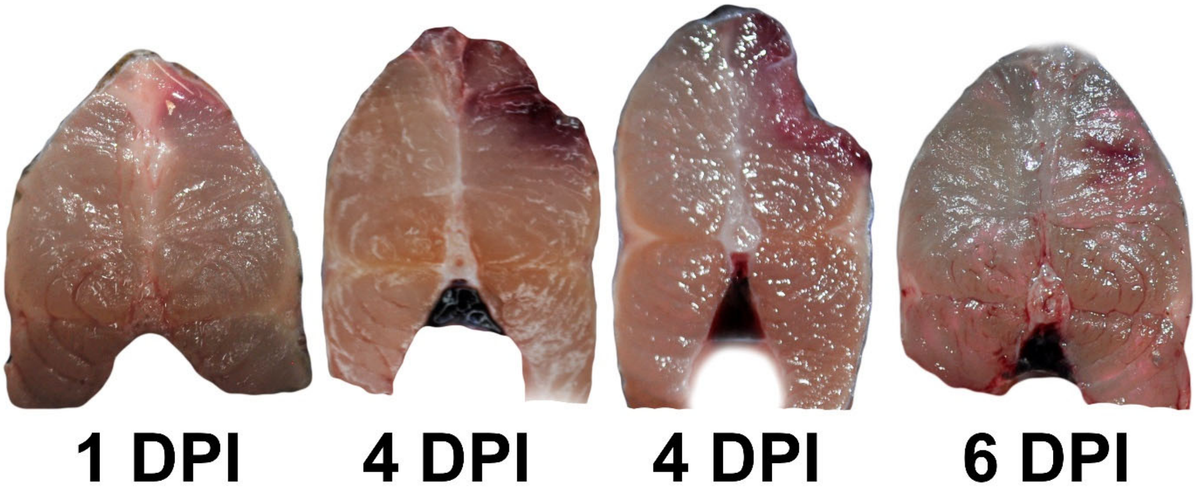

Figure S4. Condition of skeletal muscle (transverse plane) in injected rainbow trout at various experimental days.

Table S6. Hematological parameters of rainbow trout in control and infected groups throughout a chronic experiment.

Experimental group	CTR				
DPI	0	1	2	4	6
RBC, 10^6/µl	1.5±0.12	1.57±0.09	1.55±0.08	1.47±0.08	1.48±0.09
Yong erythrocyte, %	1.48±0.22	1.66±0.15	1.56±0.08	1.49±0.13	1.48±0.12
WBC, 10^4/µl	1.96±0.16	2.07±0.13	1.93±0.04	2.02±0.1	2.09±0.07
Leukocytes, %	1.85±0.28	1.93±0.45	1.88±0.28	1.76±0.2	1.96±0.18
Lymphocytes, %	93.95±0.44	94.12±0.45	90.71±1.52	94.04±0.5	93.76±0.6
Monocyte, %	2.72±0.29	1.88±0.21	4.83±0.71	2.76±0.26	2.88±0.43
Band neutorphils, %	1.49±0.31	1.88±0.21	1.63±0.28	1.52±0.26	1.41±0.2
Segmented neutorphils, %	0.63±0.08	0.98±0.06	1.19±0.4	0.54±0.05	0.77±0.13
Neutorphils sum, %	2.13±0.27	2.87±0.24	2.83±0.64	2.05±0.3	2.17±0.22
Basophils, %	1.2±0.1	1.14±0.18	1.63±0.29	1.15±0.15	1.19±0.1
Trombocytes, %	1±0.13	1.08±0.07	1.01±0.06	1.04±0.05	1.05±0.03
Experimental group	AS				
DPI	0	1	2	4	6
RBC, 10^6/µl	-	1.48±0.09	**1.37±0.05**	**1.19±0.06**	**1.31±0.06**
Yong erythrocyte, %	-	1.33±0.17	**1.55±0.11**	**1.92±0.21**	**2.23±0.1**
WBC, 10^4/µl	-	2.14±0.12	**2.26±0.15**	**2.59±0.12**	**2.26±0.07**
Leukocytes, %	-	2.26±0.46	1.71±0.39	1.98±0.14	1.99±0.22
Lymphocytes, %	-	**72.09±1.1**	**74.26±2.13**	**67.9±3**	**46.55±3.73**
Monocyte, %	-	**6.23±0.34**	**8.87±1.04**	**4.43±0.62**	3.38±0.45
Band neutorphils, %	-	**13.11±0.93**	**5.43±0.35**	**5.33±0.74**	**14.55±2.08**
Segmented neutorphils, %	-	**5.83±0.64**	**6.31±0.8**	**18.6±1.75**	**32.05±4.84**
Neutorphils sum, %	**-**	**18.94±1.03**	**11.74±0.79**	**23.93±2.2**	**46.6±4.02**
Basophils, %	-	**2.75±0.16**	**5.13±0.58**	**3.74±0.44**	**3.47±0.54**
Trombocytes, %	-	**0.95±0.05**	**0.66±0.03**	1.03±0.1	0.99±0.07

Values highlighted in bold indicate statistical significance ($p < 0.05$; $p < 0.01$) between the control (CTR) and infected group (AS) at the respective time point (0-6 DPI).

Table S8. Serum biochemical parameters of rainbow trout in control and infected group at different time points

Experimental group	CTR				
DPI	0	1	2	4	6
Bilirubin total, µmol/L	1.4±0.23	1.39±0.2	1.26±0.14	1.47±0.22	1.19±0.14
Bilirubin direct, µmol/L	0.37±0.05	0.37±0.06	0.37±0.05	0.42±0.05	0.36±0.07
AST, U/L	531.82±78.1	514.55±75.07	513.4±119.52	501.14±104.23	462.97±72.59
ALT, U/L	33.7±5.34	35.03±5.78	31.98±8.6	29.37±3.54	33.59±6.94
Urea, µmol/L	2.86±0.55	2.64±0.5	2.52±0.33	2.81±0.24	3.05±0.51
Creatinine, µmol/L	16.37±1.67	16.65±1.42	15.28±1.93	16.54±2.36	16.54±1.93
Total protein, g/L	35.5±3.56	35.71±3.79	33.07±3.24	31.65±2.7	30.78±3.06
Albumin, g/L	19.82±2.34	19.22±2.99	17.87±1.66	17.5±2.26	16.96±2.86
Alkaline phosphatase, U/L	267.83±51.99	273.2±33.5	280.73±42.61	256.95±32.15	227.63±35.89
Glucose, µmol/L	2.71±0.44	3.04±0.46	2.5±0.48	2.85±0.57	2.69±0.59
LDH, g/L	1265.5±236.35	1437.87±164.16	1127.41±207.14	1169±198.08	1300.97±206.78
Globulin, g/L	15.68±2.05	16.49±2.15	15.2±2.16	14.15±1.42	13.82±1.29
Experimental group	AS				
DPI	0	1	2	4	6
Bilirubin total, µmol/L	-	**0.47±0.05**	**0.85±0.06**	**1.22±0.05**	1.25±0.07
Bilirubin direct, µmol/L	-	**0.17±0.04**	**0.27±0.05**	**0.53±0.07**	0.43±0.05
AST, U/L	-	540.16±48.27	**714.84±81.14**	**1569.38±125.18**	**1150.51±58**
ALT, U/L	-	**20.82±1.27**	39.93±2.02	**81.48±1.49**	**60.21±5.83**
Urea, µmol/L	-	**1.58±0.2**	2.68±0.17	2.62±0.25	2.61±0.3
Creatinine, µmol/L	-	**22.72±3.52**	**19.21±2.77**	**39.35±1.6**	**29.98±0.68**
Total protein, g/L	-	35.6±3.1	41.08±5.68	28.3±2.61	27.75±1.44
Albumin, g/L	-	21.54±1.97	**23.09±1.7**	18.31±2.85	17.58±0.72
Alkaline phosphatase, U/L	-	**201.36±24.34**	**201.86±68.55**	**129.82±32.43**	203.12±16.25
Glucose, µmol/L	-	**1.39±0.31**	3.06±0.58	3.92±0.8	3.54±0.45
LDH, g/L	-	**870±100.05**	**715.35±45.45**	**1618.92±227.88**	1408.44±125.84
Globulin, g/L	-	14.06±1.56	17.98±4.17	**9.98±1.3**	**10.16±0.77**

Values highlighted in bold indicate statistical significance ($p < 0.05$; $p < 0.01$) between the control (CTR) and infected group (AS) at the respective time point (0-6 DPI).

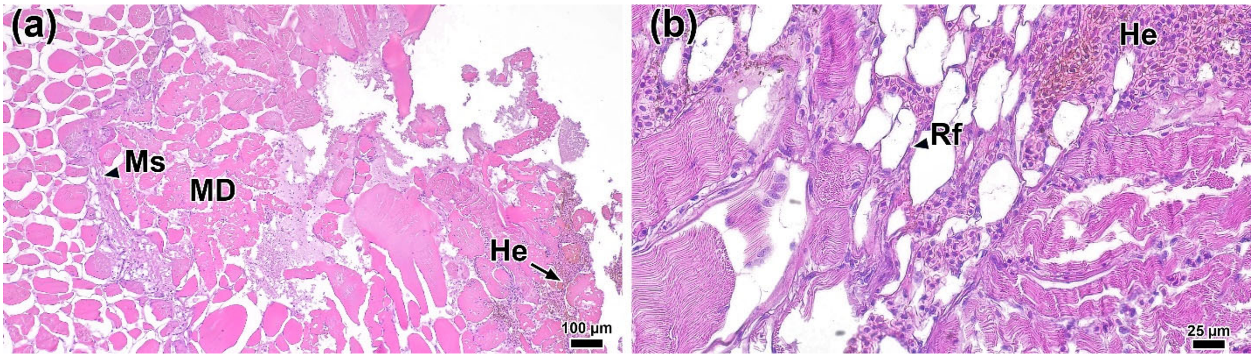

Figure S5. Histologic sections of dorsal musculature of control rainbow trout individuals at 1 (a) and 4 DPI (b). Abbreviations: MS - myosepta, MD - myomer degeneration, He - hemorrhage, RF - reticular fiber. H&E staining, scale bar 100 (a), 25 (b).

Table S9. Histomorphometric parameters of tissue elements of the posterior intestine of rainbow trout in the control and infected groups

Experimental group	CTR				
DPI	0	1	2	4	6
Mucosal epithelial height, µm	50.17±2.33	51.43±2.65	52.55±7.93	56.92±3.28	51.1±4.5
Epithelial nuclear area, µm²	47.11±3.33	52.32±3.29	47.31±1.73	45.57±5.02	51.06±5.24
Goblet cell area, µm²	117.01±13.56	130.85±10.64	116.99±13.31	110.17±5.35	115.2±10.31
Lamina propria thickness, µm	10.79±0.85	12.27±1.01	11.11±0.79	11.09±0.84	12.87±1.6
Submucosal thickness, µm	59.22±7.65	63.47±3.13	59.94±4.72	57.17±4.68	66.1±9.43
Muscularis thickness, µm	153.19±10.87	157.97±6.39	156.4±6.39	140.36±12.48	154.47±15.08
Intraepithelial leukocyte count, cells/100 µm	9.25±0.29	9.88±0.42	9.32±0.48	8.51±0.55	9.64±0.95
Goblet cell count, cells/100 µm	4.95±0.4	5.2±0.56	5.55±0.73	5.67±0.4	5.27±0.84
Experimental group	AS				
DPI	0	1	2	4	6
Mucosal epithelial height, µm	-	**46.09±3.46**	48.62±4.44	**35.39±5.7**	**42.08±4.45**
Epithelial nuclear area, µm²	-	**35.77±2.07**	43.77±7.22	**30.21±4.95**	**36.53±2.84**
Goblet cell area, µm²	-	**105.87±8.86**	137.11±18.38	**78.36±12.77**	**63.67±6.41**
Lamina propria thickness, µm	-	11.47±0.7	12.94±1.36	**23.97±2.86**	14.63±1.84
Submucosal thickness, µm	-	**56.56±3.24**	**45.32±4.4**	51.34±7.53	60.41±5.87
Muscularis thickness, µm	-	**131.42±7.71**	**119.84±12.27**	**114±17.62**	**121.33±12.75**
Intraepithelial leukocyte count, cells/100 µm	-	**11.53±0.59**	**11.71±0.67**	**11.03±1.62**	**7.89±0.98**
Goblet cell count, cells/100 µm	-	5.08±0.61	5.54±0.51	**4.58±0.73**	5.23±0.32

Values highlighted in bold indicate statistical significance ($p < 0.05$; $p < 0.01$) between the control (CTR) and infected group (AS) at the respective time point (0-6 DPI).

Table S10. Histomorphometric parameters of tissue elements of the liver of rainbow trout in the control and infected groups.

Experimental group	CTR				
DPI	0	1	2	4	6
Hepatocyte nucleus area, μm²	36.47±1.84	38.67±1.26	34.03±1.4	34.96±2.29	34.24±1.29
Hepatocyte nucleus perimeter, μm	23.36±0.8	24.71±1.07	23.19±2.14	22.56±2.46	24.24±1
Hepatocyte nucleus diameter, μm	13.55±0.25	14.91±0.45	13.88±0.92	12.37±0.62	12.91±1.03
Hepatocyte perimeter, μm	45.01±0.96	43.06±1.33	42.07±2.03	41.43±2.92	43.36±4.61
Hepatocyte area, μm²	137.19±6.52	149.3±15.48	142.28±6.95	142.63±10.97	147.09±10.32
Hepatocyte cytoplasm area, μm²	102.04±3.19	114.74±6.99	98.82±4.35	103.85±7.39	95.96±16.92
Sinusoid width, μm	4.21±0.17	4.32±0.42	4.24±0.39	4.21±0.48	4.66±0.62
Cell density, cells/100 μm²	13.48±0.48	14.17±0.74	13.71±0.72	12.58±1.36	12.74±1.36
Experimental group	AS				
DPI	0	1	2	4	6
Hepatocyte nucleus area, μm²	-	**33.25±1.31**	**37.63±1.15**	**28.89±1.62**	**26.47±2.17**
Hepatocyte nucleus perimeter, μm	-	25.4±0.44	22.45±0.59	19.84±1.21	**18.91±1.21**
Hepatocyte nucleus diameter, μm	-	14.54±1.09	13.3±0.91	**11.25±0.5**	**10.79±0.69**
Hepatocyte perimeter, μm	-	**51.88±3.22**	**46.95±0.91**	39.82±2.42	40.55±2.25
Hepatocyte area, μm²	-	132.23±17.27	**159.52±10.04**	**114.69±7.04**	**112.82±8.48**
Hepatocyte cytoplasm area, μm²	-	97.77±16.8	**120.9±8.93**	**83.22±5.65**	90.86±6.71
Sinusoid width, μm	-	**7.54±0.56**	**8.95±0.6**	**9.14±0.7**	**9.75±0.75**
Cell density, cells/100 μm²	-	**18.72±0.92**	**16.46±1.08**	**21.95±1.83**	**21.14±0.83**

Values highlighted in bold indicate statistical significance ($p < 0.05$; $p < 0.01$) between the control (CTR) and infected group (AS) at the respective time point (0-6 DPI).

Table S11. Histomorphometric parameters of tissue elements of the trunk kidney of rainbow trout in the control and infected groups

Experimental group	CTR				
DPI	0	1	2	4	6
Area of the glomerulus, µm²	1456.43±86.2	1778.16±292.38	1331.12±136.49	1387.31±105.55	1437.36±185.15
Area of the renal corpuscle, µm²	2191.96±86.68	2307.05±134.88	2125.85±396.69	2413.2±234.54	2237.97±169.12
Area of Bowman's space, µm²	840.85±115.18	897.62±194.61	734.73±165.76	819.99±118.74	755.62±90.7
Thickness of the proximal tubule epithelium, µm	12.35±0.67	14.00±0.56	12.8±1.24	12.17±0.5	11.62±0.95
Thickness of the distal tubule epithelium, µm	15.21±0.62	16.67±1.36	16.11±2.09	15.08±0.59	14.62±0.89
Experimental group	AS				
DPI	0	1	2	4	6
Area of the glomerulus, µm²	-	1836.04±259.87	**2139.8±185.55**	**2325.54±142.46**	1367.18±39.02
Area of the renal corpuscle, µm²	-	2557±203.74	**3189.95±177.45**	**3209.22±264.27**	2264.39±109.57
Area of Bowman's space, µm²	-	812.7±212.85	**1043.59±88.49**	908.71±181.91	871.09±122.13
Thickness of the proximal tubule epithelium, µm	-	13.46±1.14	13.37±1.01	11.27±0.63	**10.47±0.17**
Thickness of the distal tubule epithelium, µm	-	15.8±1.46	15.79±0.89	**16.18±0.59**	13.69±0.75

Values highlighted in bold indicate statistical significance ($p < 0.05$; $p < 0.01$) between the control (CTR) and infected group (AS) at the respective time point (0-6 DPI).

Table S12. Abbreviation of histopathological indexes and morphometric parameters used in the work.

Abbreviation	English Definition
AB	Apoptotic bodies
ALB	Albumins
ALP	Alkaline phosphatase
ALT	Alanine aminotransferase
ANI	Average nucleotide identity
AMR	Antimicrobial resistance
AS	Infected group
AST	Aspartate aminotransferase
BC	Blood congestion / Stasis
BD	Bile duct
BS	Bowman's space
BV	Blood vessel
CARD	Comprehensive Antibiotic Resistance Database
CD	Cellular debris
CI	Confidence intervals
CM	Circular muscle layer
CRE	Creatinine
CFU	Colony Forming Units
CTR	Healthy group
DBI	Direct bilirubin
dDDH	Digital DNA-DNA hybridisation
De	Desquamation
DPI	Days post-injection
DT	Distal renal tubule
ECA	Enterobacterial common antigen
El	Ellipsoid
EN	Epitheliocyte nucleus
Er	Erythrocyte
Fb	Fibrosis
FBA	Fish blood agar
GA	Glomerular arteriole
GBDP	Genome Blast Distance Phylogeny
GC	Goblet cell
GCAT	Glycerophospholipid-cholesterol acyltransferase
GL	Glomerulus
GLO	Globulins
GLU	Glucose
H&E	Hematoxylin-eosin
He	Hemorrhages
HPI	Hours Post Injection
HN	Hepatocyte nucleus
HT	Hematopoietic tissue
IEr	Immature erythrocyte

IEL	Intraepithelial lymphocytes
IF	Inflammatory focus
im	Intramuscularly
In	Infiltration
LD50	Median lethal dose
LDH	Lactate dehydrogenase
Li	Liver
LM	Longitudinal muscle layer
Ly	Lymphocyte
Ma-l	Macrophage-like cell
MD	Myomere degradation
Mm	Myomere
MMC	Melanomacrophage centers
MN	Myomere nucleus
MS	Myosepta
MT	Masson's trichrome
Mu	Muscular layer
NA	Nutrient Agar
Ne	Necrosis
NH	Necrotized hepatocyte
NNT	Neonephrogenic tubules
Oe	Edema
OD600	Optical density
PAS	Periodic acid-Schiff
PBS	Phosphate-buffered saline
PE	Parietal epithelium
PGAP	NCBI Prokaryotic Genome Annotation Pipeline
PI	Posterior intestine
PN	Pyknotic nuclei
PT	Proximal renal tubule
RC	Reticular fiber
REC	Reticuloendothelial cell
RF	Reticular fiber
RGI	Resistance Gene Identifier
RNe	Band neutrophil
RP	Red pulp
SC	Spleen capsule
SG	Stratum granulosum
SM	Submucosa
SNe	Segmented neutrophil
SP	Spleen
SRCAMB	State Research Center of Applied Microbiology and Biotechnology
SV	Supranuclear vacuoles
T2SS / T3SS / T6SS	Type II / III / VI secretion system
TBI	Total bilirubin
TP	Total protein

Tr	Trabecula
TSB	Tryptone soy broth
Tt	Trabecula
TYGS	Type Strain Genome Server
URE	Urea
УЗВ	Recirculating aquaculture system
УНУ	Unique scientific facility
Va	Vacuolization
VFDB	Virulence Factor Database
WP	White pulp
